# C_3_N nanodots inhibits Aβ peptides aggregation pathogenic path in Alzheimer’s disease

**DOI:** 10.1038/s41467-023-41489-y

**Published:** 2023-09-15

**Authors:** Xiuhua Yin, Hong Zhou, Mengling Zhang, Juan Su, Xiao Wang, Sijie Li, Zaixing Yang, Zhenhui Kang, Ruhong Zhou

**Affiliations:** 1 Institute of Quantitative Biology, Shanghai Institute for Advanced Study, College of Life Sciences, Zhejiang University, Hangzhou, 310027 China; 2 Jiangsu Key Laboratory for Carbon-based Functional Materials and Devices, Institute of Functional Nano and Soft Materials (FUNSOM), Soochow University, Suzhou, 215123 China; 3 Macao Institute of Materials Science and Engineering (MIMSE), MUST−SUDA Joint Research Center for Advanced Functional Materials, Macau University of Science and Technology, Taipa, 999078 Macao China; 4 State Key Laboratory of Radiation Medicine and Protection, School for Radiological and Interdisciplinary Sciences (RAD-X), Soochow University, Suzhou, 215123 China; 5 Department of Chemistry, Columbia University, New York, NY 10027 USA

**Keywords:** Alzheimer's disease, Cognitive ageing, Protein aggregation, Nanoparticles

## Abstract

Despite the accumulating evidence linking the development of Alzheimer’s disease (AD) to the aggregation of Aβ peptides and the emergence of Aβ oligomers, the FDA has approved very few anti-aggregation-based therapies over the past several decades. Here, we report the discovery of an Aβ peptide aggregation inhibitor: an ultra-small nanodot called C_3_N. C_3_N nanodots alleviate aggregation-induced neuron cytotoxicity, rescue neuronal death, and prevent neurite damage in vitro. Importantly, they reduce the global cerebral Aβ peptides levels, particularly in fibrillar amyloid plaques, and restore synaptic loss in AD mice. Consequently, these C_3_N nanodots significantly ameliorate behavioral deficits of APP/PS1 double transgenic male AD mice. Moreover, analysis of critical tissues (e.g., heart, liver, spleen, lung, and kidney) display no obvious pathological damage, suggesting C_3_N nanodots are biologically safe. Finally, molecular dynamics simulations also reveal the inhibitory mechanisms of C_3_N nanodots in Aβ peptides aggregation and its potential application against AD.

## Abstract (web summary)

In this work, the authors report the utilization of nano-inhibito C3N nanodots to inhibit Aβ peptides aggregation and fibrils disassembly, and show how they induce a cognitive enhancement in treated AD mice.

## Introduction

Alois Alzheimer reported the first case of Alzheimer’s disease (AD) in 1906^1,2^. Now, more than one century later, AD remains an unresolved public health problem worldwide^3^. AD is a progressive neurodegenerative disease associated with insidious onset and slow progression of behavioral and cognitive dysfunction. The severity of the AD from early stage^4^ advances to obvious symptoms which further aggravates the need to utilize immediate remedies against the progression of the disease. Moreover, the incidence of AD also increases with the increasing age reflected by the increasing rate of ~27.6% in 65–74 year-old people to ~36.4% in people over 80 years old^5^. This significant increase with age also poses a worldwide threat of acquiring AD among elderly population. This also urges the need of developing novel and effective AD management therapies for clinical purposes.

Growing evidence suggests the aggregation of Aβ peptides is highly related with synaptic dysfunction, neuroinflammation, oxidative stress damage, neurotoxicity mediated by the triggered hyperphosphorylation of downstream Tau protein, as well as the ultimate cell death^6–9^. Additionally, Aβ oligomers also drive pathology by damaging cell membranes, activating receptors, disrupting signaling, impairing mitochondria, perturbing the trans-Golgi network, inducing endoplasmic reticulum stress, causing endosomes/lysosomal leakage, and triggering macroautophagy^6,10–15^. In contrast, reversal of the Aβ peptides aggregation process also offers a suitable therapeutic strategy against AD. However, the successful implementation of this concept remains a huge challenge despite decades of effort along this direction. On the other hand, the lack of effective drugs against AD, with only two FDA-approved options available, such as aducanumab^16^ and lecanemab^17^, still raising high demand for alternate therapeutic options. Encouragingly, in a phase-III clinical trial, another monoclonal antibody agent called donanemab exhibited promising positive results^18^. Besides, other anti-AD agents (including peptides^19,20^, polymers^21,22^, small drug molecules^23–26^, and metal oxides^27^) show only a very mild inhibition effect on Aβ peptides aggregation. Recently, nanomaterials (NMs) (e.g., graphene oxide^28^, fullerenes^29,30^, quantum dots^31^, carbon nanotube^32^, and g-C_3_N_4_^33,34^) have been reported to inhibit, directly or indirectly, the aggregation of Aβ peptides, including both the inhibition of oligomer fibrillization and disaggregation of mature fiber in vitro. The potential of these NMs to inhibit aggregation is closely related to their physical and chemical properties, including size, curvature, and modifications^35,36^. But very few of them can still work in vivo. Interestingly, graphene quantum dots were also found to inhibit α-synuclein aggregation, disassociate mature fibrils, and penetrate the blood-brain barrier (BBB) leading to ultimate protection of dopamine neurons^37^. Therefore, the use of nanomaterials may offer valuable alternate source as therapeutic agents for protein conformational diseases (e.g., AD, Parkinson’s disease, Huntington’s disease, Type 2 diabetes).

In this study, we demonstrate that C_3_N nanodots can significantly inhibit Aβ peptides aggregation and disaggregate mature Aβ fibrils, relieve aggregation-induced neuron cytotoxicity, rescue neuronal death, protect neurites from damage, and exhibit only mild cytotoxicity both in vitro and in vivo. Moreover, the intraperitoneal administration of C_3_N nanodots for 6 months significantly improves the learning and spatial memory abilities of APP/PS1 in double transgenic AD mice. Additionally, the underlying molecular mechanism of Aβ peptide aggregation inhibition by C_3_N nanodots has also been explored using all-atom molecular dynamics (MD) simulations. Thus, we believe our current study provides deep insights into the anti-Aβ peptides aggregation capability of C_3_N nanodots and its potential application against AD.

## Results

### C_3_N nanodots inhibit Aβ_42_ peptides fibrillization in vitro

**Fig. 1:**
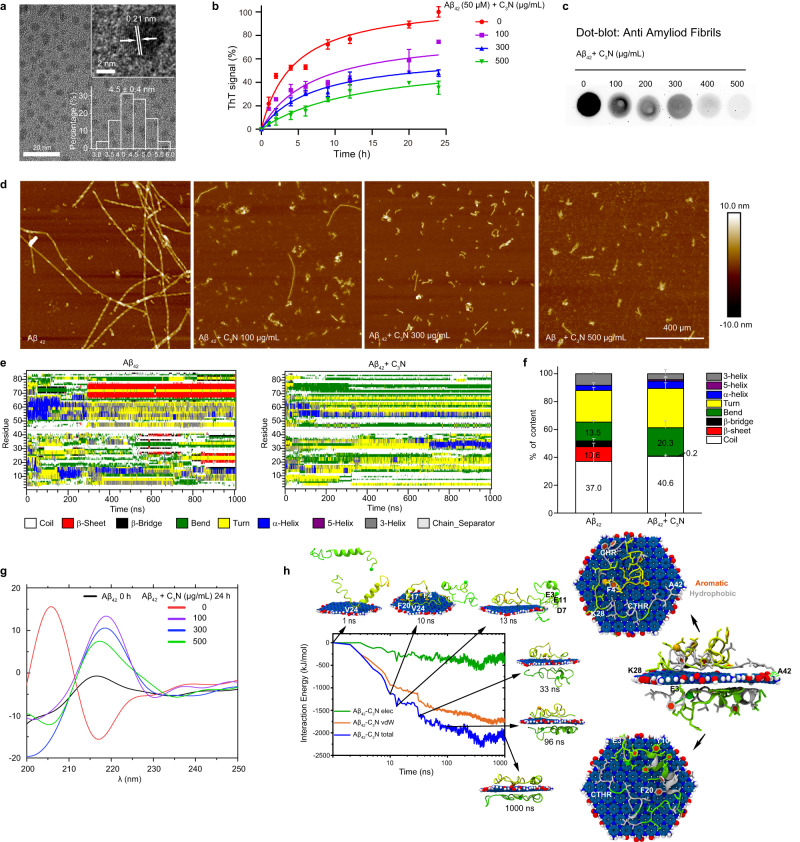
C_3_N nanodots inhibit Aβ_42_ fibrillization in vitro. **a** Transmission electron microscopy (TEM) image, crystal structure (top right corner, HRTEM image), and lateral size distribution (bottom right corner, histogram) of C_3_N nanodots. The image is representative of three independent experiments. **b** The influence of C_3_N nanodots on Aβ_42_ peptides (50 μM) aggregation was detected by ThT fluorescence. Data are presented as mean ± SD, *n* = 3 biological replicates and signals were normalized by setting the maximal ThT signals to 100%. **c** The formation levels of amyloid fiber under different conditions were detected by dot blot assay using Aβ fibrils conformation specific antibody (mOC87), at time = 24 h. Immunoblots are from one experiment representative of three independent experiments with similar results. **d** Representative AFM images of Aβ peptides untreated/treated with C_3_N nanodots (0, 100, 300, and 500 μg/mL) for 24 h. *n* = 3 independent experiments. **e** Time evolutions of the secondary structure of each residue in two Aβ_42_ peptides. The secondary structures of residues were assigned using the DSSP definition^72^. **f** The proportions of each structural component in the peptides. **g** CD spectra of Aβ peptides at 0 and 24 h in the absence of C_3_N nanodots and after incubation with C_3_N nanodots for 24 h. **h** The nonbonded interaction energies (including electrostatic (elec), van der Waals (vdW) interactions, and a total of them) between C_3_N nanodots and peptides and key binding configurations during the process. Green dashed lines indicate hydrogen bonds, and the hydrophobic and hydrophilic (polar/charged) residues are shown with silver and green, respectively. Source data are provided as a Source data file.

C_3_N nanodots were synthesized by polymerization of 2,3-diaminophenazine using hydrothermal synthesis following a previous report^38^. The synthesized nanodots had an average lateral size of 4.5 ± 0.4 nm (Fig. 1a) with a lattice spacing of 0.21 nm, which corresponds to the (100) plane of graphite. Meanwhile, these nanodots had a height of less than 1 nm, indicating a stacking arrangement of one or two layers (Supplementary Fig. 1). Initially, the identification and characterization of C_3_N nanodots were performed using several spectroscopic techniques including UV–visible (UV–Vis) absorption spectroscopy, Fourier transform infrared (FTIR) spectroscopy and X-ray photoelectron spectroscopy (XPS). (Supplementary Fig. 2).

We first studied the role of C_3_N nanodots towards the aggregation behavior of Aβ_42_ peptides, which were shown to have more implications than Aβ_40_ in forming neurotoxic assemblies and causing AD pathogenesis^39,40^. In the absence of C_3_N nanodots, Aβ_42_ peptides aggregated into mature amyloid fibers, as demonstrated by various experimental procedures. This included the utility of ThT fluorescence, dot blot assay, atomic force microscope (AFM), transmission electron microscope (TEM), and CD spectroscopy. During these investigations, C_3_N nanodots effectively inhibited the aggregation of Aβ_42_ peptides (Fig. 1). It was evident from delayed aggregation kinetics and reduced ThT fluorescence intensity (after convergence of aggregation process) following C_3_N nanodots treatment. The inhibition strength was found positively correlated with C_3_N nanodots treatment concentration (Fig. 1b). It should be noted that, under the concentrations examined, C_3_N nanodots did not entirely inhibit the aggregation of peptides. The final peptide self-assembly samples were also examined through dot blotting using an amyloid fiber conformation-specific antibody (mOC87)^41^. Notably, amyloid fiber content decreased with the increasing concentration of C_3_N nanodots during treatment (Fig. 1c). This confirmed the inhibition function of C_3_N nanodots against peptides aggregation. Morphologically, Aβ_42_ peptides aggregated to long and well-defined mature fibers after 24 h in PBS without C_3_N nanodots, as demonstrated through AFM and TEM imaging (Fig. 1d and Supplementary Fig. 3). In contrast, incubation with C_3_N nanodots for 24 h resulted in a gradual morphologic change of Aβ_42_ peptides self-assembly samples from long mature fibers to diffused punctiform structures. Furthermore, it is worth noting that the aggregation of N-truncated Aβ peptides (AβpE3) and Aβ_40_ is also likely to contribute to the molecular pathology of AD. Our investigation also delved into the impact of C_3_N nanodots on the aggregation of these two peptide species. Remarkably, comprehensive evaluations encompassing ThT fluorescence, dot blot assay, CD spectra, TEM, and AFM unequivocally demonstrated the potent ability of C_3_N nanodots to effectively impede the aggregation of these peptides (Supplementary Figs. 4 and  5).

To our surprise, C_3_N nanodots exhibited an exceptional capacity to disassemble mature fibrils of Aβ_42_ as well. The convergence of evidence from ThT fluorescence, dot blot assay, CD spectra, AFM, and end-to-end distance results collectively substantiate that, in a concentration and duration-dependent manner, the co-incubation of mature fibrils with C_3_N nanodots led to the gradual dismantling of these originally long, well-defined fibrils into smaller, amorphous entities (Supplementary Fig. 6). Overall, these results suggested that C_3_N nanodots effectively reverse the aggregation of Aβ peptides.

To further unveil the regulating process and underlying molecular mechanisms of C_3_N nanodots towards inhibiting aggregation of these peptides, we then performed all-atom molecular dynamics (MD) simulations (Supplementary Fig. 7). In the absence of C_3_N, two Aβ_42_ peptides self-assembled into a partially ordered structure (containing β-sheets). However, C_3_N nanodot application significantly inhibited the formation of any β-sheets. For instance, in two out of three trajectories (run 1 and run 3), very rare β-sheet contents were formed (i.e., in run 2, β-sheet appeared at t = 80 ns, then disappeared at t = 340 ns) (Fig. 1e and Supplementary Fig. 8). Convergence of the simulations (>900 ns) demonstrated an overall decrease in the β-sheet content of ~10.6 ± 1.5% without C_3_N to 0.2 ± 0.6% with C_3_N. Simultaneously, the random-coiled and bend components increased from ~37.0 ± 2.4% to ~40.6 ± 1.7%, and ~13.5% to ~20.3% (Fig. 1f), respectively. These findings suggested that C_3_N nanodot effectively redirects Aβ_42_ peptides self-assembly to disordered structures. Moreover, CD spectroscopy confirmed that C_3_N nanodots redirected the secondary structure of Aβ_42_ peptides (at time = 24 h) from the β-sheet-rich to disordered random-coiled conformations (Fig. 1g). These results sufficiently demonstrate the structural modulating role of C_3_N nanodot in impeding the aggregation of Aβ_42_ peptides.

The detailed interaction energies including both van der Waals (vdW) and electrostatic (elec) interactions between C_3_N and peptides were also explored (Fig. 1h). This was performed by analyzing the key binding configurations in a typical trajectory to better illustrate the binding mechanisms. Driven by vdW and hydrophobic interactions, one peptide was adsorbed onto the surface of C_3_N (time = 1 ns) and strengthened by π‒π stacking interactions (F4 and F20) (time = 10 ns). At time = 13 ns, another peptide was adsorbed onto the edge of C_3_N by electrostatic attractions between E11, D7, and E3 residues with ‒NH_3_^+^ groups at the edge of C_3_N nanodot. At time = 33 ns, this peptide was fully adsorbed onto the other side of C_3_N nanodot via vdW and π‒π stacking interactions. After 96 ns, the adsorption process converged. At this state, most hydrophobic and aromatic residues were adsorbed onto the C_3_N nanodot surface. Meanwhile, some charged or polar residues formed salt-bridge or hydrogen bonds with edge groups (e.g., ‒COO^‒^ and ‒NH_3_^+^) of C_3_N nanodot while suppressing subsequent aggregation of peptides. Hence, the strong adsorption between peptides and C_3_N nanodot was collectively driven by a combination of vdW and electrostatic, hydrophobic, hydrogen bonding, and π‒π stacking interactions, with the vdW interaction dominating (Fig. 1h), to induce disruption in peptides self-assembly and form disordered structures.

Moreover, we conducted a comparative analysis of the inhibitory effects of stacked C_3_N nanodots (two layers), nano graphite (GRA) (simulated by two layers of stacked graphene), and fullerene (e.g., C_60_) on the aggregation of Aβ_42_ peptides. The findings clearly indicate that C_3_N nanodots exhibit a relatively stronger capability in inhibiting Aβ peptide aggregation compared to the other two alternatives (Supplementary Fig. 9). Notably, the electrostatic potential (ESP) calculations of C_3_N nanodots reveal the presence of numerous polar C–N bonds and charged edge groups (e.g., ‒COO^‒^ and ‒NH_3_^+^), resulting in a significantly polar surface for C_3_N nanodots (Supplementary Fig. 10), which significantly differs from GRA and fullerene represented by Lennard–Jones (LJ) particles. These distinct surface properties of C_3_N nanodots enable more effective suppression of peptide aggregation through multiple interactions, including vdW, electrostatic, hydrophobic, hydrogen bonding, and π‒π stacking interactions. Furthermore, these surface properties confer advantages upon C_3_N nanodots, such as superior water dispersity and compatibility with cell membranes, in contrast to highly hydrophobic candidates like nano GRA, fullerene, and others.

### C_3_N-nanodots alleviate neuron cytotoxicity induced by Aβ_42_ peptides and demonstrate superior cytocompatibility

**Fig. 2:**
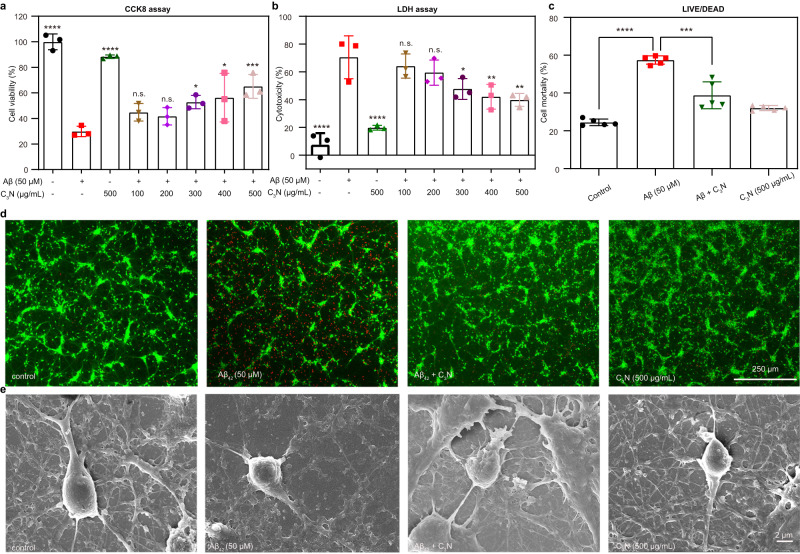
C_3_N nanodots reduce Aβ_42_-aggregation-induced cytotoxicity. Neurons were cultured with/without Aβ_42_ peptides for 24 h. Cytotoxicity of Aβ_42_ aggregates in the presence/absence of different concentrations of C_3_N nanodots for 24 h to primary neurons was assayed by **a** CCK8 (*P* < 0.0001, *P* < 0.0001, *P* = 0.0300, 0.0114, and 0.0009, respectively) and **b** LDH-release (*P* < 0.0001, *P* < 0.0001, *P* = 0.0312, 0.0065, and 0.0035, respectively), *n* = 3 independent experiments. Statistical significance was determined by one-way ANOVA in (**a**–**c**) with *p* < 0.05 considered statistically significant. **c**, **d** Live/dead staining experiments to examine whether C_3_N nanodots alleviate the cytotoxicity of neurons induced by Aβ_42_ peptides. *n* = 5 independent experiments. Statistical significance was determined by unpaired Student’s *t* test (two-tailed) with *P* < 0.05 considered statistically significant (*P* < 0.0001, *P* = 0.0005). **d** Photomicrographs of live/dead assay showing live (green cell body) and dead (red nuclei) cells in each group. **e** Morphology of cells in each group was observed under SEM. The images are from one experiment representative of three independent experiments with similar results. All data are presented as mean ± SD. *P < 0.05, **P < 0.01, ***P < 0.001, and ***P < 0.0001 *vs* 50 μM Aβ_42_ group. n.s. = not significant. Source data are provided as a Source data file.

As shown above, C_3_N nanodots exhibited an effective inhibiting function against Aβ_42_ peptides aggregation at molecular level. At this stage, it was logical to examine whether C_3_N nanodots alleviate aggregation-induced neuron cytotoxicity (Fig. 2). Herein, we analyzed primary neuron cells viability and toxicity under different conditions using cell counting kit 8 (CCK-8), Lactate Dehydrogenase (LDH), and Live/Dead assays. The CCK-8 assay results demonstrated that Aβ_42_ peptides aggregation causes severe toxicity in neurons. This was found after neuronal cells incubation with 50 μM Aβ_42_ peptides for 24 h which resulted in a survival rate of only ~29.89 ± 3.98%. However, increased treatment concentration with C_3_N nanodots resulted in improved cell survival rate: ~44.83 ± 6.90% (100 μg/ml) to ~65.52 ± 9.12% (500 μg/mL) (Fig. 2a). Hence, C_3_N nanodots dose-dependently relieved Aβ_42_ peptides aggregation-induced neuron cytotoxicity, which was further confirmed by LDH (Fig. 2b) and Live/Dead experimental (Fig. 2c, d) results. In addition, the cytotoxicity of C_3_N nanodots was found very mild with C_3_N nanodots administered at 500 μg/mL resulting a neuronal survival rate of ~88.47 ± 1.36%. We further investigated the morphologies of neurons under different conditions using scanning electron microscope (SEM) technology (Fig. 2e). Normal neurons presented in a plump-pear shape with many dendrites. However, Aβ_42_ aggregation-induced significant deformations of neurons, e.g., the cellular body shrunk notably and was accompanied by severe dendrites loss. In contrast, the treatment with C_3_N nanodots resulted in well maintained dense dendrites suggesting inverse effect against the toxicity caused by Aβ_42_ peptides aggregation in neurons. It also distinguished the mild influence of C_3_N nanodots on the shape of neurons. These effects predominantly stem from the fact that C_3_N nanodots facilitate the reversal of Aβ_42_ peptide aggregation. Additionally, the adsorption of peptides onto the surface of C_3_N nanodots, leading to a decrease in peptide concentration in the solution, is expected to play a role in alleviating the cytotoxicity of Aβ_42_ peptides to neurons. It is noteworthy that both the drug and peptide concentrations employed at the cellular level are relatively high. As we transition to the animal level, attaining elevated drug concentrations requires surmounting the BBB, a challenge that could potentially be addressed through sustained and long-term administration strategies.

In addition, the cytotoxicity of C_3_N nanodots in several cell lines was also examined, including red blood cells (RBCs), primary mouse neuron (Neuron), rat adrenal chromaffin cell tumor cells (PC12), primary rat astrocyte (Astrocyte), human umbilical vein endothelial cells (HUVECs), human neuroblastoma cells (sh-sy5y). The results showed that C_3_N nanodots possess decent cytocompatibility among all tested cell lines (Supplementary Fig. 11 and Supplementary Fig. 12). Moreover, C_3_N nanodots showed much superior biocompatibility than GO nanosheets (Supplementary Fig. 13). This revealed that C_3_N nanodots alleviate neuron cytotoxicity, reduce cell death, protect Aβ_42_ aggregation-induced axonal and dendritic damages and demonstrate remarkable cytocompatibility.

### C_3_N-nanodots improve the learning and spatial memory capabilities of APP/PS1 mice with limited biotoxicity

**Fig. 3:**
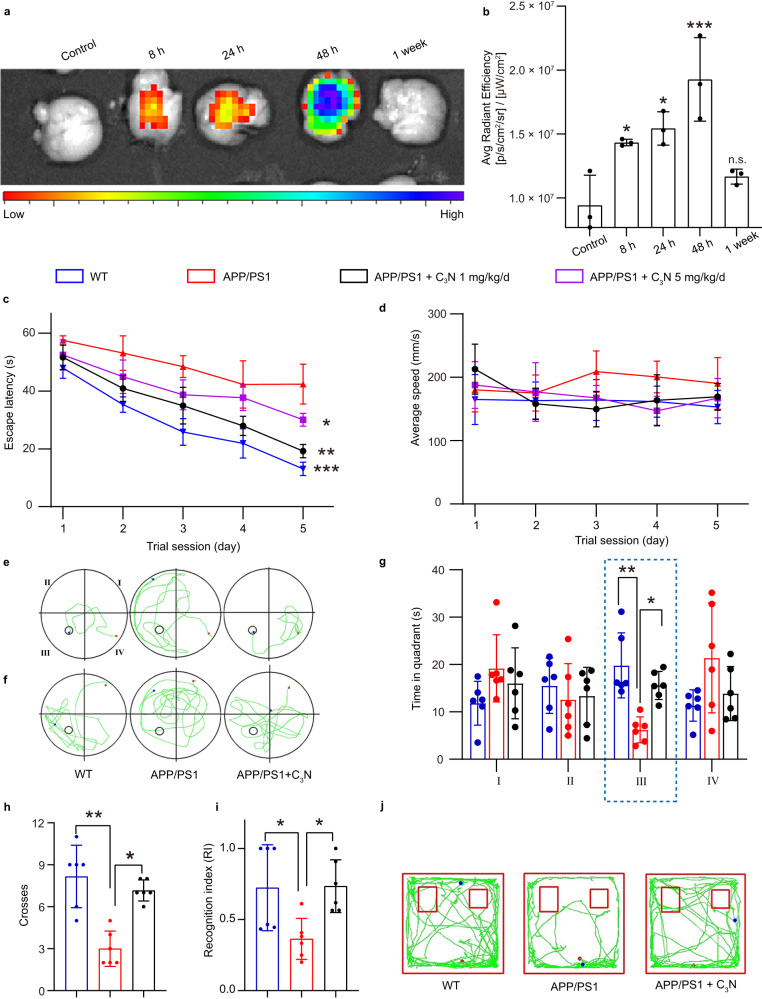
C_3_N nanodots rescues the cognition deficits of the APP/PS1 mice. **a**, **b** Temporal changes in fluorescence intensity of C_3_N-Cy5.5 in the mouse brain following intraperitoneal injection (i.p.) at a relatively high dosage of 200 mg/kg to ensure optimal imaging. *n* = 3 mice per group. The *P* value represents the significant difference between the C_3_N nanodots-treated groups and the control group determined by one-way ANOVA (*P* = 0.0331, 0.0106, and 0.0003, respectively). **c** Time to reach hidden platform in Morris water maze of the WT and APP/PS1 mice treated without/with C_3_N nanodots (Two-way ANOVA for groups, *P* = 0.0396, 0.0019, and 0.0001). **d** The average swimming velocity of each group. **e** Representative swimming paths of escape latency in the fifth day. **f** Representative 60 s swimming paths of mice treated with various regimens to locate the escape platform after platform retrieval. **g** Accumulated time spent by mice treated with different regimens in all four quadrants. (*P* = 0.0016 and 0.0257). **h** Frequency of mice traversing the platform position after platform retrieval (*P* = 0.0024 and 0.0209). **i** The novel object recognition index (RI) of mice in each group mice (*P* = 0.0311 and 0.0168). **j** Representative paths of novel object recognition. All data are presented as mean ± SD. n.s. = no significants, *n* = 6 mice each group. Statistical significance was determined by one-way ANOVA in (**g**–**i**) with *P* < 0.05 considered statistically significant. *P < 0.05, **P < 0.01 and ***P < 0.001. Source data are provided as a Source data file.

Following the encouraging in vitro findings, we sought to determine whether C_3_N nanodots have neuroprotective functions towards AD mice via inhibition of Aβ peptides aggregation. For this purpose, we used APP/PS1 double transgenic mice as the model AD organism. Here, male mice were chosen exclusively for this study as they may have a relatively stable hormone level and much less estrogen’s impact^42–44^. It thus allows for a more accurate observation and evaluation of disease progression and pathological changes upon the application of nanomedicine. This in-vivo model overexpresses Aβ peptides in the brain by inducing amyloid plaque formation which eventually leads to the occurrence of AD symptoms^45,46^. The expression of Aβ peptides in APP/PS1 mice begins at 3‒4 months of age. Thus, we treated the APP/PS1 mice with C_3_N nanodots-saline solution per day from 3 to 9 months via intraperitoneal injection. APP/PS1 mice received saline only were set as the positive control group, and wild-type (WT) mice with non-intervention were set as the negative control. After six months of C_3_N nanodots injection vs. no injection, the cognitive function of APP/PS1 mice were examined using the Morris water maze and novel object recognition tests (Fig. 3).

In order to further ascertain the ability of C_3_N nanodots to traverse the BBB and accumulate within the brain, an essential prerequisite for their potential application in AD treatment, we employed Cy5.5-modified C_3_N nanodots (referred to as C_3_N-Cy5.5) to enable fluorescence imaging. For this purpose, we conducted experiments utilizing healthy C57BL/6J mice, which were divided into five distinct groups: (1) a control group without any treatment, and (2) groups that received intraperitoneal (i.p.) administration of C_3_N-Cy5.5 nanodots for 8 h, (3) 24 h, (4) 48 h, and (5) 1 week. The administration of C_3_N-Cy5.5 nanodots was accomplished via injections of a PBS solution at a relatively high dosage of 200 mg/kg (*n* = 3 per group) to ensure optimal imaging. As shown in Fig. 3a, b, at time = 8 h, the fluorescence emanating from C_3_N-Cy5.5 nanodots within the brain was discernible. Subsequently, the highest intensity of fluorescence was observed at 48 h post-injection, gradually diminishing to undetectable levels after one week. These compelling outcomes substantiate the remarkable capacity of C_3_N nanodots to successfully penetrate the BBB, thus establishing a crucial foundation for their potential therapeutic application in AD.

Then, we refined the optimal C_3_N nanodots administration dose from the assessment of the escape latency. In the Morris water maze test during the 5-day learning phase, the latency time for APP/PS1 mice to find the survival platform (initially placed in the third quadrant) in the saline group underwent a very mild decrease from ~57.5 ± 1.5 to ~42.4 ± 6.9 s as shown previously^47^. Treatment with C_3_N nanodots significantly shortened the latency time, indicating a remarkably improved learning capacity of AD mice (Fig. 3c). We also noted that treatment with 1 mg/kg/d dose obtained better therapeutic effect than that treated with 5 mg/kg/d dose, at the day 5 the latency time was ~19.2 ± 2.3 s vs. ~29.3 ± 2.9 s (Fig. 3c), suggesting that 1 mg/kg/d may be the optimal dose. The potential contribution of the swimming capability (swimming speed) to the learning effects was excluded because there was no distinct difference in the average swimming speed between two C_3_N nanodots treated groups and the WT mice (Fig. 3d). Overall, these results demonstrated the efficacy of C_3_N nanodots in the treatment and improving the learning capacity of AD mice, with an optimal dose of ~1 mg/kg/d. Hence, 1 mg/kg/d was used in the following in vivo experiments.

To further measure the spatial memory capability, the third quadrant residence time of mice was accumulated during 60 s swimming after retrieval of the survival platform on day 6 (Fig. 3e, f). C_3_N nanodots-treated AD mice spent significantly more time in the third quadrant and crossed this target quadrant more often compared to control APP/PS1 mice (~15.9 ± 2.8 s vs. ~6.5 ± 2.5 s; ~7.2 ± 0.8 times vs. ~3.0 ± 1.3 times) (Fig. 3f, g, & h). In addition, the time to explore the new object among APP/PS1 mice was significantly reduced as compared to that of the WT mice (~0.7 ± 0.3 vs. ~0.4 ± 0.1). However, treatment with C_3_N nanodots can remarkably prolong the time of APP/PS1 mice to explore the new object, which resulted in the recognition index (RI) of APP/PS1 mice (treated with C_3_N nanodots) was remarkably improved to level comparable with WT mice (~0.7 ± 0.3 vs. ~0.7 ± 0.2) (Fig. 3i, j). These results were indicative that C_3_N nanodots treatment could partially rescue these defects in APP/PS1 mice and may offer utility against AD.

Furthermore, the body weights among both C_3_N nanodots treated and untreated AD mice increased steadily during the entire administration period (Supplementary Fig. 14) suggesting the higher biocompatibility of C_3_N nanodots in animals. Moreover, the H&E staining in heart, liver, spleen, lung, and kidney tissue showed no distinct lesions (Supplementary Fig. 15). We also noted in the literature that GO-based nanomaterials have the ability to reverse the aggregation of Aβ and α-synuclein peptides^28,37^. However, it is worth pointing out that numerous studies have raised concerns about their potential long-term cytotoxicities, including inflammation reactions^48–50^. In light of these concerns, we conducted measurements of several inflammation markers after six months of treatment with C_3_N nanodots. Remarkably, all investigated inflammation indexes, such as white blood cell count (WBC), lymphocyte count (Lymph#), monocyte count (Mon#), and granulocyte count (Gran#), fell within the normal healthy range (Supplementary Fig. 16). These findings strongly indicate that C_3_N nanodots do not provoke severe inflammation reactions. Additionally, the biodistribution of C_3_N nanodots suggests that the liver and kidney were the primary off-target organs of C_3_N nanodots (Supplementary Fig. 17). Consequently, we examined liver and kidney function indicators, such as aspartate aminotransferase (AST), albumin (ALB), and urea (UREA), and found no significant differences in these function indices (Supplementary Fig. 18). This further supports the exceptional biocompatibility of C_3_N nanodots. Taken together, these toxicological assessments collectively suggest that C_3_N nanodots exhibit minimal toxicity in vivo.

Moreover, we performed the investigation of the excretion pathways of C_3_N nanodots and discovered that urination and defecation played vital roles in their elimination from the body (Supplementary Fig. 19). On the other hand, degradation studies conducted under simulated physiological conditions, including an acidic environment similar to lysosomes and the presence of catalase with physiological concentrations of H_2_O_2_, revealed the degradability of C_3_N nanodots (Supplementary Figs. 20 and 21). Cell colocalization experiments further confirmed the entry of C_3_N nanodots into lysosomes (Supplementary Fig. 21), implying their potential decomposition through cellular lysosome degradation mechanisms. This inherent bio-degradable property may confer enhanced bioavailability and biosecurity to C_3_N nanodots, highlighting their potential as a biocompatible and safe candidate.

### In vivo efficacy of C_3_N nanodots against amyloid pathology

**Fig. 4:**
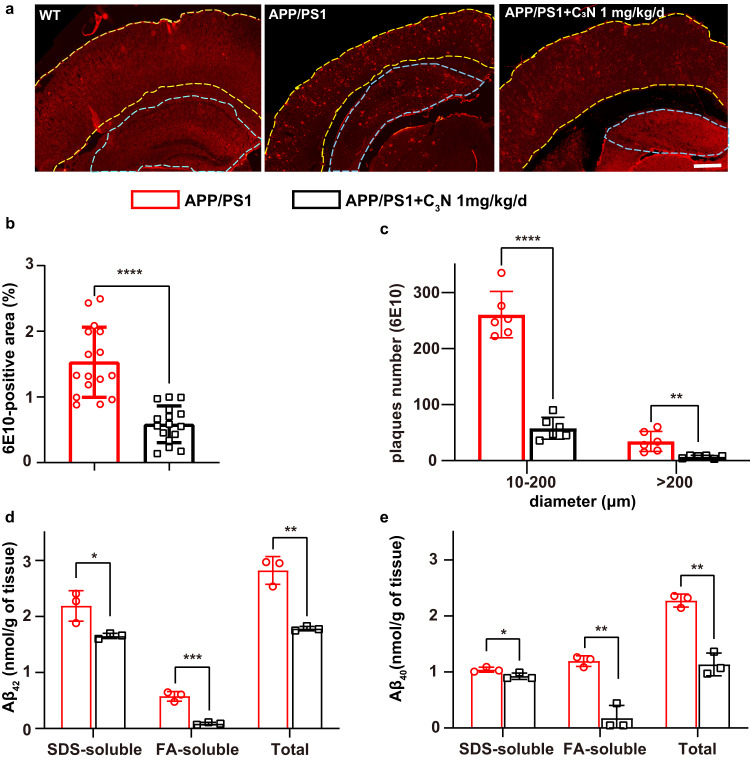
C_3_N nanodots reduce Aβ deposition levels in the brain of APP/PS1 mice. After six months of treatment, the whole brains of APP/PS1 mice treated with/without C_3_N nanodots were collected. **a** 6E10-labeled mice brain sections immunostained for Aβ (6E10) and showing the amyloid plaque levels of the WT and APP/PS1 mice under different conditions. The cortex and hippocampus regions are marked with yellow and blue dashed lines, respectively. Scale bar = 500 μm. **b** 6E10-positive area (*n* = 16 images over 3 mice per group, *P* < 0.0001) and **c** number of 6E10-positive plaques in different sizes (*n* = 6 images over 3 mice per group, *P* < 0.0001, *P* = 0.0041) in the AP*P*/PS1 mice untreated/treated with C_3_N nanodots at the doses of 1 mg/kg/d, respectively. **d**, **e** Levels of Aβ_42_/Aβ_40_ peptides in SDS‒, FA‒, and TBS‒ soluble forms in the cortex, *n* = 3 mice per group, *P* = 0.0285, 0.0007, 0.0021, 0.0498, 0.0021, and 0.0011 respectively. Statistical comparisons were performed between the APP/PS1 and C_3_N nanodots-treated groups, according to the Student’s *t*-test (two-tailed). Da*t*a are presented as mean ± SD.*P < 0.05, **P < 0.01, ***P < 0.001 and ****P < 0.0001 *vs* APP/PS1 group. Source data are provided as a Source data file.

Next, we detected the level of cerebral fibrillar amyloid plaques as hallmark of AD^9^ in WT and APP/PS1 mice untreated/treated with C_3_N nanodots. The 6E10 anti-Aβ antibody was used because of its specific binding capability with residues 1 to 16 of the Aβ peptide. Notably, massive amyloid plaques accumulated in both the cerebral cortex and hippocampus of APP/PS1 mice treated with saline (~1.5 ± 0.5%) (Fig. 4a). However, the amyloid plaques deposition levels remarkably decreased after treatment with 1 mg/kg/d (~0.6 ± 0.3%; a ~60% decrease) C_3_N nanodots treatment (Fig. 4b). These results were also confirmed by counting the number of amyloid plaques (Fig. 4c).

Considering that Aβ_40_ and Aβ_42_ peptides are the dominant component of the plaques in the brains of AD patients^39^. We then used enzyme-linked immunosorbent assay (ELISA) to quantify the level of intra-cephalic Aβ_42_/Aβ_40_ peptides. This involved using Tris-buffered saline (TBS)‒, sodium dodecyl sulfate (SDS)‒, and formic acid (FA)‒soluble Aβ forms corresponding to the soluble, partially soluble (non-dense plaque), and completely insoluble (dense plaque) Aβ forms, respectively. These analyses showed that treatment with C_3_N nanodots decreased the level of total Aβ_42_/Aβ_40_ peptides by ~36%/~50%, respectively. The relative FA‒soluble Aβ_42_ / Aβ_40_ species levels was reduced most significantly by ~84%/~83% (Fig. 4d, e), which suggested that C_3_N nanodots effectively inhibit Aβ peptides aggregating into completely insoluble dense plaques. Overall, C_3_N nanodots possessed the strong ability to delay or obstruct Aβ peptide aggregation pathogenesis in vivo.

### C_3_N nanodots improve the level of synaptic function-related proteins in vivo

**Fig. 5:**
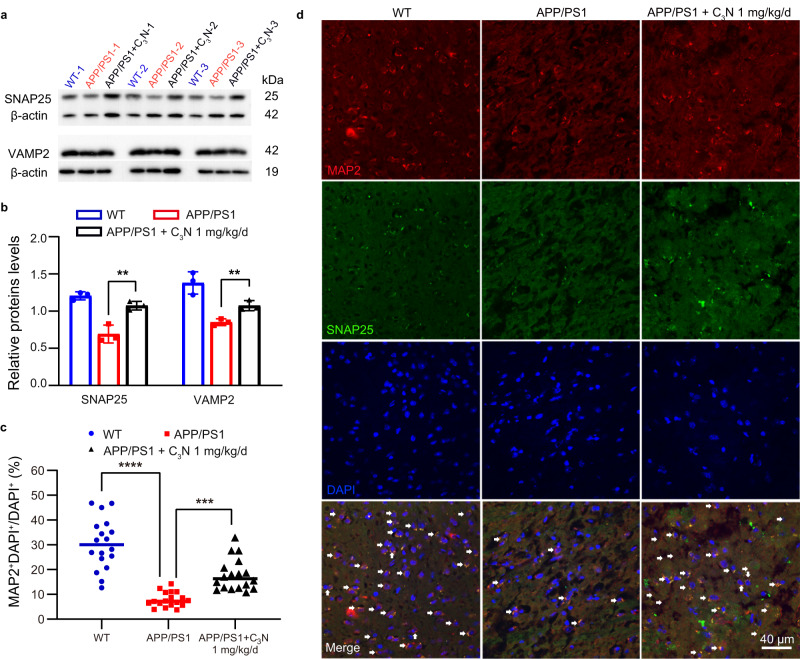
C_3_N nanodots increase the expression levels of the synaptic function-related proteins in APP/PS1 mice. **a** SNAP25 and VAMP2 proteins levels were assessed using western blotting. **b** The relative expression levels of the SNAP25 and VAMP2 proteins were estimated by comparing their relative gray densities to the β-actin. *n* = 3 mice per group, Statistical significance was determined by unpaired Student’s *t* test (two-tailed) with *P* < 0.05 considered statistically significant. *P* = 0.0077 and *P* = 0.0075. **c** Quantitation of MAP2-positive neurons in cortexes. *n* = 18 micrographs examined over 3 independent mice. Statistical significance was determined by one-way ANOVA in with *P* < 0.05 considered statistically significant. *P* < 0.0001, *P* = 0.0002. **d** Immunohistochemistry on brain sections of different group mice. Representative micrographs of MAP2-labeled (red), SANP25-labeled (green) and DAPI (blue) in the cortex. Micrographs from three independent mice with similar results. All experiments were repeated three times. Data are presented as mean ± SD. **P < 0.01, ***P < 0.001 and ****P < 0.0001 *vs* APP/PS1 group Source data are provided as a Source data file.

Synaptic dysfunction is another important pathological feature of AD^51,52^ having a strong impact in nerves development and neurotransmitters release (including dopamine and glutamate). The SNAP25 and VAMP2 proteins are the two main synaptic proteins which protects synaptic integrality^53,54^. Therefore, we assessed the changes in expression levels of these two proteins using western blot and immunohistochemistry fluorescence assays. Western blot results demonstrated that the content of two proteins was up-regulated (Fig. 5a) after treatment with C_3_N nanodots. The quantification of the SNAP25 and VAMP2 protein levels was performed using gray density analyses by utilizing Image J software. The results showed that expression levels of the two proteins were increased by 43% & 22% respectively, after treatment with C_3_N nanodots (Fig. 5b).

In addition, we also examined the neuron number and synaptic damage by double-staining brain tissue with an antibody against microtubule-associated protein 2 (MAP2; a neuronal marker) and SNAP25 (a synaptic marker) (n = 3/group). The co-localization of SNAP25 and MAP2 signals reflected the expression level of synaptic proteins in neurons. Remarkably, C_3_N treatment preserves MAP2-positive neuron numbers in APP/PS1 mice exposed to Aβ (Fig. 5c, d), demonstrating a ~2.3-fold upregulation. In APP/PS1 mice treated with saline only, SNAP25/MAP2 co-localization yellow pixel intensity decreased remarkably, which indicated serious dysfunction of the neural network as compared to WT mice. In contrast, treatment with C_3_N nanodots for six months resulted in significantly elevated SNAP25/MAP2 expression levels (Fig. 5d). These results demonstrated that C_3_N nanodots maintains an effective protective function in the synapse.

## Discussion

In this study, an effective Aβ peptides aggregation nano-inhibitor called C_3_N nanodots has been explored against AD. This nano-inhibitor redirects peptide self-assembly to disordered off-pathway species and disassembly mature fibrils into smaller and amorphous entities, thereby reducing aggregation-induced neuron cytotoxicity in vitro and in vivo. Several experimental analyses including ThT fluorescence, dot blot assays and CD spectra collectively demonstrate that C_3_N nanodots guide Aβ_42_, AβpE3, and Aβ_40_ peptides self-assembly to disordered structures rather than β-sheet-rich structures. Similarly, morphological observations using AFM and TEM imaging show that after treatment with C_3_N nanodots these Aβ peptides form small diffused oligomeric structures, in contrast to the long and well-defined mature amyloid fibers formed in the absence of C_3_N nanodots. Moreover, ThT fluorescence, dot blot assay, CD spectra, AFM, and end-to-end distance results collaborate that C_3_N nanodots can disaggregate the preformed long, well-defined mature fibrils into smaller, amorphous species. The results from CCK-8, LDH, and Live/Dead assay reveal that C_3_N nanodots relieve neuron toxicity induced by Aβ aggregation and rescue neuronal death. SEM images further helped to depict that C_3_N nanodots protect normal neuronal morphology from Aβ aggregation-induced destruction. Furthermore, MD simulations demonstrated that both the non-specific hydrophobic and electrostatic interactions, and the specific π‒π stacking and hydrogen bonding interactions between C_3_N nanodots and Aβ peptides synergistically obstruct the aggregation process of Aβ peptides. The inhibitory capability of C_3_N nanodots on peptides aggregation is notably superior to that of GRA and fullerene. This can be attributed to the polarization of the SEP of C_3_N nanodots, which is induced by the presence of numerous polar C‒N bonds and charged edge groups (e.g., ‒COO^‒^ and ‒NH_3_^+^). These characteristics enable C_3_N nanodots to engage in additional electrostatic interactions with the charged residues within the amyloid peptides, distinguishing them from GRA and fullerene represented by LJ particles. Moreover, the formation of hydrogen bonds between the charged edge groups of C_3_N nanodots and peptides contributes to the suppression of peptides aggregation.

Fluorescence imaging experiments provide evidence that C_3_N nanodots are capable of effectively crossing the BBB in mice and accumulating in the brain. The cognitive abilities among the studied mice were also restored following C_3_N nanodots treatment. After C_3_N nanodots treatment, the cognitive ability of APP/PS1 mice significantly improved to levels comparable with WT mice. Several immunological experiments including immunohistochemistry fluorescence, western blot, and ELISA assays demonstrated that APP/PS1 mice experience a decrease in cerebral fibrillar amyloid plaque levels and an increase in SNAP25 and VAMP2 with C_3_N nanodots treatments. Furthermore, rigorous toxicological assessments, including changes in body weight, H&E staining of vital organs (heart, liver, spleen, lung, and kidney), long-term inflammation indexes, and liver and kidney function indicators, demonstrate the exceptional biocompatibility of C_3_N nanodots. The main excretion pathways of C_3_N nanodots were found to be through urination and defecation. Additionally, simulated degradation studies suggest that C_3_N nanodots may undergo degradation via cellular lysosome and catalase degradation pathways. This may further enhance the bioavailability and biosecurity of C_3_N nanodots. Conclusively, this study not only provides useful experimental and theoretical basis for the application of C_3_N nanodots in neuronal protection, but also offers the groundwork for subsequent optimal designs of nanomaterials targeting Aβ peptides aggregation in AD.

## Methods

### Preparation of C_3_N nanodots and characterizations

The synthesis of C_3_N nanodots was based on the method reported by our group^38^. Briefly, the aqueous solution of 2,3-Diaminophenazine (80 mL, 1.4 mM) was heated and kept at 320 °C for 36 h in a 100 mL poly (p-phenylene)-lined stainless-steel autoclave. The products were filtered by 0.02 µm alumina microporous membrane to obtain the raw C_3_N nanodots. Then, the raw C_3_N nanodots were treated with H_2_O_2_ (5 M, 80 °C for 6 h) for further oxidization. Finally, the sample was purified via membrane dialysis with the molecular weight cutoff of 50–1000 Da for 5 days, and the oxygen-modified C_3_N nanodots were obtained.

The transmission electron microscopy (TEM) and high-resolution TEM (HRTEM) images were obtained using transmission electron microscope with the accelerating voltage of 200 kV (Tecnai G2 F20, FEI Corporation, American). The Fourier transform infrared (FT-IR) spectra of C_3_N nanodots were characterized using fourier transform infrared spectrometer (Hyperion, Bruker Corporation, Germany). The UV–Vis spectra analysis utilized a UV-vis spectrophotometer (Lambda 750, PerkinElmer, American), and the X-ray photoelectron spectra (XPS) were obtained using an X-ray photoelectron spectrometer (Axis ultra DLD, Kratos, Britain).

### Preparation of Cy5.5-conjugated C_3_N nanodots

To achieve fluorescence labeling of the C_3_N nanodots, a reaction was carried out by incubating the resulting covalent C_3_N nanodots with the Cy5.5 monofunctional N-Hydroxysuccinimide ester (Cy5.5-NHS) in PB buffer at pH 8.0. This incubation process was allowed to proceed overnight, facilitating the successful conjugation of Cy5.5 dye to the C_3_N nanodots through covalent bonding. Following the reaction, any unreacted Cy5.5 dye was eliminated through the utilization of ultrafiltration. The resultant product, namely Cy5.5-conjugated C_3_N nanodots (denoted as C_3_N-Cy5.5), was subsequently stored in a dark environment at a temperature of 4 °C for future applications.

### Antibodies

Primary antibodies for immunoblotting including dot blot analysis and western blot analysis were performed with antibodies against Amyloid Fibril-Conformation-Specific (mOC87, abcam, Cat#: ab201062, 1:8000), SNAP25 (Synaptic systems, Cat#: 111-002, 1:2000), VAMP2 (abcam, Cat#: ab3347, 1:1000), β-actin (4D3, Bioworld Technology, Cat#: BS6007M, 1:5000). Secondary antibodies were conjugated with peroxidase affinipure donkey anti-rabbit IgG (H + L) (#711-035-152, Jackson ImmunoResearch, 1:10,000), Peroxidase affinipure donkey anti-mouse IgG (H + L) (#715-035-151, Jackson ImmunoResearch, 1:10,000).

Primary antibodies for immunohistochemistry were directed against purified anti-β-Amyliod 1-16 (6E10, Covance, SIG-39320, 1:500); MAP2 (AP20, Millipore, Cat#: MAB3418, 1:1,000) and SNAP25 (Synaptic systems, Cat#: 111-002, 1:2,000). Secondary antibodies were conjugated with Cy^TM^3 affinipure donkey anti-mouse IgG (H + L) (#715-165-151, 1:400), Alexa Fluor® 488 affinipure donkey anti-rabbit IgG (H + L) (#711-545-152, 1:400).

### Preparation of monomic Aβ_42_ peptides

**Synthetic** Aβ_42_ (NH2-DAEFRHDSGYEVHHQKLVFFAEDVGSNKGAIIGLMVGGVVIV-COOH, purity ≥ 98%), AβpE3 (NH2-Pyr-FRHDSGYEVHHQKLVFFAEDVGSNKGAIIGLMVGGVVIV-COOH, purity ≥ 98%) and Aβ_40_ (NH2-DAEFRHDSGYEVHHQKLVFFAEDVGSNKGAIIGLMVG GVV-COOH, purity ≥ 98%) peptides were purchased from APeptide Co., Ltd (Shanghai, China) and prepared according to protocols previous described^55,56^. Briefly, Aβ_42_/Aβ_40_/AβpE3 peptides were first dissolved in hexafluoroisopropanol (HFIP, 10522, Sigma Aldrich) and sonicated for 10 min. The Aβ_42_‒HFIP solution was then incubated at room temperature for 1 h to ensure the monomerization and structural randomization of peptides, and placed into a fume hood to completely evaporate HFIP. The obtained peptide film was stored at ‒80 °C. Immediately before use, the peptide film was resuspended to 5 mM in dimethyl sulfoxide (DMSO, D2650, Sigma Aldrich) and diluted to a final concentration of 100 μM in phosphate-buffered solution (PBS, 0.1 M). The solution was then centrifuged at 16,000 × *g* for 10 min at 4 °C to remove the pre-formed fibers.

In the aggregation experiment, Aβ_42_ (100 μM) was mixed with C_3_N nanodots at various concentrations or PBS solution to a final concentration of 50 μM and then incubated at 37 °C with constant agitation at 300 rpm for 24 h.

### Preparation of Aβ_42_ fibrils

Aβ_42_ peptide stock solutions were prepared by dissolving them in DMSO and phosphate buffer (pH = 7.4) to achieve a final concentration of 200 μM. The peptides were then aggregated for 48 h at 37 °C and centrifuged at 16,000 × *g* for 10 min to remove insoluble material. The concentration of the stock solutions was determined using the Bradford assay. Subsequently, the peptide stock solutions were diluted in 1 mM phosphate buffer (pH = 7.4) to a final concentration of 50 μM. The samples were incubated with different concentrations of C_3_N nanodots at 37 °C with continuous shaking at 300 rpm. The ability of C_3_N nanodots to disaggregate mature fibrils was assessed using the ThT fluorescence assay, Dot blot assay, CD spectra, and AFM images.

### Thioflavin-T (ThT) assay

Fluorescence with Thioflavin T (ThT) was used to detect aggregated Aβ containing β-sheets^57^. A 50 μL sample was mixed with 150 μL ThT (20 μM, T3516, Sigma Aldrich) in a 96-well plate. The resulting fluorescence intensity was detected immediately after mixing with a fluorescence plate reader (BioTek, USA) at excitation and emission wavelengths of 450 nm and 485 nm, respectively. Fluorescence values of C_3_N nanodots and ThT were subtracted from that of the mixed solution. Error bars (±s.d.) of triplicate samples are shown for selected data points.

### Dot blot assay

Dot blot assays were carried out with amyloid fibril conformation specific antibody to probe the formation level of Aβ_42_ amyloid mature fibers. Briefly, 5 μL aliquots of the sample were dropped onto nitrocellulose membranes (1060002, GE Healthcare). Once the membranes dried, they were blocked for 1 h with 3% nonfat milk in tris-buffered saline (TBS) solution and then incubated with Anti-Amyloid Fibril antibody (mOC87) overnight at 4 °C. The membranes were washed 3 times in TBST for 5 min and then incubated with the horseradish peroxidase (HRP)-conjugated donkey anti-rabbit secondary antibody for 2 h at room temperature (Fig. 1a and Supplementary Figs. 4b, 5b, 6b). Finally, the membranes were developed by chemiluminescence using ECL Plus (P0018S, Beyotime).

### Atomic force microscope (AFM)

Here, 10 μL of each sample was dispersed on freshly cleaved mica sheets. After air-drying, samples were scanned and analyzed using the tapping mode of AFM (Bruker, Germany), and the height of the sample was recorded.

### Transmission electron microscopy (TEM)

Ten microliters of each sample were dispersed on a copper grid (carbon and formvar coated 300 mesh, Zhongjing Technology Co., Ltd, China) for 2 min at room temperature. Then, they were washed twice with ultrapure water and negatively stained with 1% uranyl acetate for 2 min. After air-drying, images of peptides were observed using a Tecnai G2 spirit BioTwin TEM at 120 kV.

#### Circular dichroism (CD) spectroscopy

All samples were diluted six times under PBS conditions. Spectra were detected using a Jasco J-815 circular dichroism spectropolarimeter (1 mm path length cuvette) at 25 °C. The spectrum of PBS was set as the baseline. Each sample was scanned three times and the average value was adopted. Raw data, after subtracting the buffer spectra, were smoothed according to the manufacturer’s instructions.

### Primary neuron cultures

Mouse primary cortical neurons were obtained from embryonic day 18 C57BL/6J mice. All animal procedures followed the policies of the Soochow University Animal Care and Use Committee (SUACUC). In brief, dissociated neurons were plated onto dishes coated with poly-D-lysine (P6407, Sigma Aldrich) then suspended in culture medium (Neurobasal Media (21103-049, Invitrogen) containing 2% B-27 (17504-044, Invitrogen), 1% penicillin/streptomycin (15140122, P/S, Gibco), 1% l-glutamine and 0.25% GlutaMax^TM^ (35050, Invitrogen)). Next, the plating medium was substituted with feeding medium (Neurobasal medium supplemented with 2% B27, 1% P/S, and 1% l-glutamine) on the second day after cell plating. The medium was replaced twice a week and the cultures were incubated in a 5% CO_2_ incubator at 37 °C. Cells were used for experimentation 8 days after seeding.

### Primary astrocyte cultures

Primary astrocyte cultures were extracted from the cerebral cortex of 1-3-d-old rats (Sprague-Dawley). In brief, dissociated cortical cells were suspended in DMEM media (sh30022.01b, Hyclone) containing 1% P/S (Gibco) and 10% Fetal bovine serum (10099141, Gibco) and plated on PDL-coated 75 cm^2^ flasks at a density of 6 × 10^5^ cells/cm^2^. Monolayers of type 1 astrocytes were harvested 12–14 days after plating. Non-astrocytic cells were separated and removed from the flasks by shaking and changing the medium. Astrocytes were dissociated through trypsinization and reseeded on uncoated 96-well plates. The cells grew to 80–90% confluence before exposure to C_3_N nanodots.

### In vitro cytotoxicity study

The cytotoxicity of C_3_N nanodots was assessed using a standard CCK-8 assay (CK04, Dojindo). Primary mouse neurons, rat adrenal pheochromocytoma cells (PC12, CRL-1721, purchased from ATCC), primary rat astrocytes, human umbilical vein endothelial cells (HUVCEs, PCS-100-013, purchased from ATCC), and human neuroblastoma cells (sh-sy5y, CRL-2266, purchased from ATCC) were selected for the study. Briefly, cells in the logarithmic growth phase were seeded at a density of 5 × 10^3^ cells per well in 96-well plates and cultured in complete DMEM medium (#11965092, Gibco) containing 10% FBS (#03.U16001DC, EallBio) and 1% penicillin/streptomycin (#15140163, Gibco) at 37 °C with 5% CO_2_. The cells were then co-cultured with various concentrations of C_3_N nanodots (0, 50, 100, 150, 200, 300, 400, and 500 μg/mL) in serum-free DMEM medium until they reached approximately 80% confluence. After a 24-h incubation period, the cells were washed three times with PBS. The CCK-8 assay was performed according to the manufacturer’s instructions. The absorbance (optical density, OD) of cells in different groups was measured at 450 nm using a microplate reader (Bio-Tek Instruments, Synergy NEO, USA) to calculate the cell viability using the following equation:

1 $${{{{{\rm{cell\; viability}}}}}}(\%)=(({{{{{{\rm{OD}}}}}}}_{{{{{{\rm{test}}}}}}}-{{{{{{\rm{OD}}}}}}}_{{{{{{\rm{blank}}}}}}})/({{{{{{\rm{OD}}}}}}}_{{{{{{\rm{control}}}}}}}-{{{{{{\rm{OD}}}}}}}_{{{{{{\rm{blank}}}}}}}))\times 100\%$$

where, OD_test_ refers to the absorbance of the cells exposed to the nanomaterial sample, OD_control_ refers to the absorbance of the control sample, and OD_blank_ refers to the absorbance of the blank well. Each sample was tested in five replicates.

To compare the cytotoxicity between C_3_N nanodots and GO nanosheets, a standard CCK-8 assay was performed using mouse brain microvascular endothelial cells (bEnd.3, cl-0598, purchased from Procell), BV2 murine microglial cells (BV2, cl-0493, purchased from Procell), and HUVCEs. GO was purchased from TimeNano (product model: TNWGO-3; more characterizations were provided in our previous literature^58^). The cells were co-cultured with different concentrations of C_3_N nanodots or GO nanosheets (0, 62.5, 125, 250, and 500 μg/mL) in serum-free DMEM medium for 24 h. The aforementioned standard protocol was then followed.

### Evaluation of C_3_N nanodots relieve the neurotoxicity of Aβ_42_ oligomers

Cytotoxicity of Aβ_42_ oligomers on primary neuron was evaluated using CCK-8 kit, LDH cytotoxicity assay kit (K311-400, Biovision), and Live/Dead kit (l3224, Invitrogen). Before experimentation, the neuron culture medium was used to dilute 5 mM Aβ_42_ peptide stock solution and C_3_N nanodots solution to achieve a mixture of 50 μM Aβ_42_ and C_3_N nanodots at various concentrations (e.g., 100, 200, 300, 400, and 500 μg/mL). A control group with medium solution and experimental groups with 50 μM Aβ_42_ peptide solution and 500 μg/mL C_3_N nanodots solution were analyzed. The culture solutions were incubated at 4 °C for 24 h and then added to cells for another 24 h at 5% CO_2_ humidified environment 37 °C.

The LDH assay was performed according to LDH cytotoxicity assay kit instructions. A group of cells treated with 1% Triton X-100 was added as a positive control; the cell-free group was the negative control. Optical density at 490 nm was measured on a microplate reader and the cytotoxicity of each group was calculated according to:

2 $${{{{{\rm{cytotoxicity}}}}}}(\%)=	 ({{{{{\rm{Test\; Sample}}}}}}-{{{{{\rm{Negative\; Control}}}}}}) \\ 	 /({{{{{\rm{Positive\; Control}}}}}}-{{{{{\rm{Negative\; Control}}}}}})\times 100\%$$

For the Live/Dead assay, the prepared dye was incubated with cells for 15 min according to the Live/Dead kit instructions. Cells were then photographed under a fluorescence microscope (Leica, Germany), and live vs. dead cells were counted using Image J software.

### Morphology observation of primary neuron

Primary neurons were planted on cell culture slides, washed twice with PBS, and fixed overnight with 2.5% glutaraldehyde at 4 °C for morphological observation. Twenty-four hours later, they were washed 3× with ultrapure water for 5 min each. Then, 30%, 50%, 70%, 80%, 90%, 95%, and 100% ethanol dehydration occurred in sequence for 10 min. Gold was then sprayed on the surface of the sample, and cell morphology was observed using a scanning electron microscope (SEM, Zeiss, Germany).

### Animals and drug treatment

APP/PS1 [B6C3-Tg (APPswePSEN1dE9)/Nju] double transgenic AD mice and C57BL/6J mice were used in this study (Nanjing Model Animal Research Center, Nanjing, China). All experiments were reviewed and approved by the Animal Ethics Committee of Soochow University (Nos.: SUDA201807A422 and SUDA201907A025). APP/PS1 mice were produced and maintained on a C57BL/6J hybrid background with free access to chow and drinking water under a 12-h light/dark cycle under constant temperature (22 ± 1 °C) and humidity (40‒70%).

Only male mice were tested in this study. APP/PS1 mice were randomly divided into three groups. The positive control group was intraperitoneally (i.p.) injected with vehicle (saline; APP/PS1 group). The other two groups were injected intraperitoneally with either 1 mg/kg or 5 mg/kg C_3_N nanodots solution. Littermate WT mice treated with saline solution were used as negative controls (WT group). Drugs were given once per day from 3 months of age for six months.

### Biodistribution study

Healthy male C57BL/6J mice of 6 months old were selected for ex vivo fluorescence (FL) imaging to verify the biodistribution of C3N-Cy5.5 nanodots. The C57BL/6J mice were purchased from from the SLACCAL Lab Animal Ltd (Shanghai, China) and maintained on C57BL/6J background. The experiment was reviewed and approved by the Animal Ethics Committee of Soochow University (No.: SUDA202007A648). The C57BL/6J mice were sacrificed at 8 h, 24 h, 48 h, and 1 week after i.p. injection of C_3_N-Cy5.5 nanodots, administered at a dosage of 200 mg/kg (*n* = 3 per group). The main organs were collected for FL imaging and semiquantitative biodistribution analysis. The distribution of C_3_N-Cy5.5 nanodots was tracked using an IVIS Spectrum Imaging System (PerkinElmer, USA), and FL imaging was performed at specific time points after the injections. The excitation wavelength used was 680 nm, and the emission wavelength was 710 nm.

### Excretion study

The C57BL/6J mice were purchased from the SLACCAL Lab Animal Ltd (Shanghai, China) and maintained on C57BL/6J background. The experiment was reviewed and approved by the Animal Ethics Committee of Soochow University (No.: SUDA202007A648). Three healthy male C57BL/6J mice of 6 months old were intraperitoneally injected with Cy5.5-labeled C_3_N nanodots at a dosage of 100 mg/kg. To monitor the excretion and distribution of the nanodots, each mouse was individually placed in a metabolic cage to facilitate the collection of urine and feces at predetermined time intervals. Following each collection, the metabolic cage was thoroughly washed and disinfected to ensure cleanliness and prevent cross-contamination. The collected urine and feces samples were carefully preserved at a temperature of −80 °C until further analysis. To determine the concentration of C_3_N-Cy5.5 nanodots in the metabolites, the samples were subjected to measurement using Cy5.5 fluorescence. The results were then expressed as the percentage of the injected dose per gram/milliliter of feces/urine, providing insights into the excretion dynamics and distribution patterns of the nanodots.

#### Evaluation of intracellular biodegradation of C_3_N nanodots

C_3_N nanodots (1 mg/mL) were dissolved in 0.3 M acetate buffer at a pH of 5.0, creating an acidic condition similar to lysosomes. The resulting solution was incubated in a shaker at 120 rpm and 37 °C. The absorbance of the solution was continuously monitored to evaluate the acid-responsiveness of the C_3_N nanodots. The degradation rate (R) of the C_3_N nanodots was determined using the following equation:

3 $${{{{{\rm{R}}}}}}=({{{{{{\rm{A}}}}}}}_{0}-{{{{{{\rm{A}}}}}}}_{{{{{{\rm{t}}}}}}})/{{{{{{\rm{A}}}}}}}_{0}\times 100\%$$

Where A_0_ represents the initial absorbance value (OD#) of the solution at 0 h, and A_t_ represents the absorbance value at time point t (t from 0–48 h).

### The biodegradation of C_3_N nanodots in vitro

The in vitro degradation behaviors of C_3_N nanodots in biomimetic microenvironments were investigated. H_2_O_2_ is typically present in the bio- microenvironment at a physiological concentration ranging from 50 × 10^−6^ to 100 × 10^−6^ M. Additionally, catalase, a common enzyme found in neutrophils, which are the main components of blood in the liver, was included in the study. To perform the experiment, C_3_N nanodots (1 mg/mL) and catalase (200 μg/mL) in 0.01 mol/L PBS (pH = 7.00) were transferred into a vial. The resulting mixture had a total volume of 20 mL and was incubated at 37 °C in the dark for 24 h. Subsequently, H_2_O_2_ (500 μmol/L) was added to initiate the biodegradation process. The sample was placed on a magnetic stirrer and subjected to constant shaking at 220 rpm. To compensate for H_2_O_2_ consumption, an additional 200 μL of H_2_O_2_ (500 μmol/L) was added each day. After 14 days of degradation, the sample was collected for transmission electron microscopy (TEM) measurement, allowing for the evaluation of structural changes and degradation effects.

### In vitro cellular uptake

The mouse brain endothelial cell line, bEnd.3 (Procell, China), was seeded at a density of 2 × 10^5^ cells per well in glass bottom cell culture dishes (Nest, 801001). The cells were cultured in high glucose DMEM medium supplemented with 10% FBS and 1% penicillin-streptomycin at 37 °C in a 5% CO_2_ atmosphere. After overnight incubation at 37 °C, the cells were treated with C_3_N-Cy5.5 at a concentration of 1 mg/mL for 5 h. PBS-treated cells were used as the negative control. Subsequently, the culture medium was replaced with Hochest 33342 dye (KeyGEN DIO tech, KGA212-50) and lysosome tracker (Invitrogen, L7526), and the cells were further incubated for an additional 20 min. After washing twice with PBS, the cells were observed using a confocal microscope (Olympus, FV1300, Japan). The Hochest 33342 channel (λex = 405 nm and λem = 460 nm), lysosome tracker channel (λex = 504 nm and λem = 511 nm), and Cy5.5 channel (λex = 640 nm and λem = 668 nm) were chosen to visualize the cell nuclei and the uptake of Cy5.5-labeled C_3_N, respectively.

### Tissue preparations

After behavioral tests, each group of mice was subdivided into two additional groups. In the first group, mice were subjected to cardiac perfusion under deep anesthesia and perfused with PBS and 4% paraformaldehyde (PFA, 158127, Sigma Aldrich) dehydrated with sucrose. Simultaneously, the major organs of the mice were meticulously harvested at each designated time point, followed by fixation in neutral buffered formalin (10%). Subsequently, the specimens were subjected to routine processing, wherein they were embedded in paraffin and sectioned into 8 µm slices. These sections were stained utilizing the standard hematoxylin and eosin (H&E) protocol, and their examination was conducted under a microscope. In the second group, blood samples were collected from the mice through the extraction of ocular blood. To perform hematological analysis, 100 μL of the collected blood samples were carefully transferred into anticoagulant tubes, allowing for routine blood analysis. The remaining blood samples were kept at a temperature of 4 °C for a duration of 4 h. Following this, the blood samples were subjected to centrifugation, enabling the separation of blood serum, which was subsequently utilized for conducting blood biochemistry analysis. Mouse brains were harvested by decapitation, then quickly placed in ‒80 °C for the extraction of brain proteins.

### Western blotting analysis

Brain tissues were homogenized in cold lysis buffer (P0013C, Beyotime) containing protease inhibitor cocktail (4693116001, Roche) and centrifugated 12,000 rpm for 15 min. Supernatants were collected and the protein concentration was determined by the BCA protein assay kit (P0009, Beyotime) measured with a microplate reader. The supernatants were mixed with 5× loading buffer (#FD006, Fdbio science) incubated at 100 °C for 10 min. Each protein (15 μg) was separated by electrophoresis using a 12% SDS-PAGE gel (P0692, Beyotime) and transferred onto a PVDF membrane (ipvh00010, Millipore). The membranes were blocked by incubation with 5% non-fat milk (wt/vol) in Tris-buffered saline containing 0.1% Tween-20 (vol/vol) (TBST) for 60 min (Fig. 5a). The membranes were then incubated overnight with primary antibody (β-actin, SNAP25, VAMP2) at 4 °C. The membranes were washed thrice in TBST for 5 min and incubated with corresponding HRP–conjugated IgG secondary antibody for 2 h at room temperature (RT). The membranes were washed in TBST (3 × 5 min) before a 2-h incubation with HRP-linked secondary antibodies to rabbit or mouse accordingly at room temperature. The membranes were then visualized using chemiluminescence on ECL Plus. For the antibodies incubated in the same blots, after imaging, the blots were stripped with stripping buffer (25 mM Glycine and 1% SDS in ddH2O, pH 2.0) for 20 min at RT to remove antibodies and washed in TBST for 10 min three times. The blots were blocked at RT for 2 h in 5% non-fat milk blocking buffer in TBST and then incubated with another primary antibody. For protein quantification, densitometry was performed with ImageJ and normalized to β-actin.

### Behavioral analysis

Spatial learning and memory performance were tested using the MWM task and the novel object recognition test. The Morris water maze was conducted in a circular pool (120 cm diameter) divided into four quadrants. In the center of the third quadrant (i.e., the target quadrant), a circular platform (i.e., survival platform) with a diameter of 10 cm was placed just below the water surface (1 cm). Mice were trained four times a day for the first five days, with quadrant one as the water entry point. The time for mice to find the survival platform within 60 s was recorded. On the sixth day, the survival platform was removed, and the time spent in each quadrant and locomotion of the mice were recorded.

For the novel object recognition test, a cube (side length of 50 cm) was used, and two identical objects (i.e., old objects) were placed symmetrically at a position 10 cm from the sidewall. Mice were placed with their backs to the objects from the perpendicular bisector of the two objects, and the exploration time of the mice was recorded for 7 min. Before placing the next mice, the chamber was cleaned with 75% ethanol. The mice were trained for three days. On the fourth day, one of the old objects was replaced with a novel object and the exploration time and path were recorded. The results are represented by the novel object recognition index (RI), which was calculated as follows:

4 $${{{{{\rm{RI}}}}}}=({{{{{\rm{time}}}}}}\, {{{{{\rm{to}}}}}}\, {{{{{\rm{explore}}}}}}\, {{{{{\rm{the}}}}}}\, {{{{{\rm{new}}}}}}\, {{{{{\rm{object}}}}}}) / ({{{{{\rm{time}}}}}}\, {{{{{\rm{to}}}}}}\, {{{{{\rm{explore}}}}}} \,{{{{{\rm{the}}}}}}\, {{{{{\rm{new}}}}}}\, {{{{{\rm{object}}}}}} \\ {{{{{\rm{+}}}}}}\, {{{{{\rm{time}}}}}}\, {{{{{\rm{to}}}}}}\, {{{{{\rm{explore}}}}}} \,{{{{{\rm{the}}}}}}\, {{{{{\rm{old}}}}}}\, {{{{{\rm{object}}}}}}) \times 100\%$$

Data acquisition utilized detection and analysis software of Shanghai Xinsoft Information Technology Co., Ltd.

### Immunohistochemistry

After sucrose dehydration, brain tissue was embedded with optimal cutting temperature compound (OTC, 4583, SAKURA) and sliced into 15 μm sections (CM1950, Leica, Germany). Purified anti-β-Amyloid 1-16 (6E10) was used to examine the extracellular Aβ deposits, anti-MAP2 and anti-SNAP25 were used to detect dysfunction in neuronal networks. Brian sections were stained with primary antibodies overnight at 4 °C in a humid chamber, after being washed in PBS, followed by 2 h of incubation of Cy3-conjugated or/and 488-conjugated secondary antibodies in the dark at room temperature. Fluorescent images were acquired using a fluorescence microscope (Leica, Germany) or a confocal microscope (FV1200, Olympus, Japan) following coverslipping. The number and the area of senile plaques were quantitatively analyzed by Image J software. For histopathology of major organs, the heart, liver, kidney, spleen, lung, and kidney were isolated and stained with an H&E staining kit (ab245880, Abcam).

### Aβ_40_/Aβ_42_ quantification

Aβ_40_/Aβ_42_ content was measured using enzyme-linked immunosorbent assay (ELISA). The right hemisphere was weighed and homogenized in TBS (pH 7.4, 1:12, w/v) containing a complete protease inhibitor cocktail and centrifuged. Afterward, the precipitation was centrifuged in 2% SDS and 70% formic acid. The FA-soluble fraction was neutralized with 1 M Tris (pH 11.0) and then diluted with PBS. TBS-soluble and SDS-soluble fractions were directly diluted with PBS. Quantitation was performed according to the instructions using a Human Aβ_40_/Aβ_42_ Elisa Kit (E-EL-H0542/ E-EL-M0068km, Elabscience Biotechnology). The optical density of the samples was measured with a microplate reader (BioTek, USA) at 450 nm wavelength, and the content of Aβ_40_/Aβ_42_ in the brain was calculated as moles per gram of wet tissue.

### Statistical analysis

All results are expressed as mean ± standard deviation (SD) from at least three independent experiments. The number of mice, experiments, and statistical tests are shown for each figure in the figure legend. Statistical analyses conducted using GraphPad Prism (version 9.0) and origin (version 9.0). Datasets with only two independent groups were analyzed for statistical significance using unpaired, two-tailed Student’s *t* test. Datasets with more than two groups were analyzed using one-way ANOVA. Datasets with two independent factors were analyzed using two-way ANOVA, followed by Tukey’s post hoc test. All *p* values below or equal to 0.05 were considered significant. *P < 0.05, **P < 0.01,***P < 0.001, ****P < 0.0001.

### Simulation model system setup

The C_3_N used in the simulations had a diameter of ~4.5 nm corresponding to the average diameter of C_3_N measured in the experiments (Supplementary Fig. 7 and Supplementary data 1). The initial Aβ_42_ peptide crystal structure was taken from RCSB Protein Data Bank (PDB ID: 1Z0Q)^59^ (Supplementary Fig. 7). To investigate the effect of C_3_N on Aβ_42_ aggregation, two Aβ_42_ peptides were simulated in the absence or presence of C_3_N. In the system without C_3_N (control system), two peptides were solvated into a 9.6 nm × 9.1 nm × 6.5 nm water box containing 17,911 water molecules. The peptides + C_3_N system was derived from its counterpart, by randomly adding a C_3_N with a minimum distance of 1.5 nm to any heavy atom of the peptide. Then, two Aβ_42_ peptides + C_3_N were solvated into a water box (9.6 nm × 9.1 nm × 8.2 nm) containing 22,604 water molecules. Na^+^ and Cl^‒^ ions were added to the solvent to neutralize systems and mimic the physiological conditions of 0.15 mol/L NaCl. In addition, we also compared the inhibitory effects of stacked C_3_N (two layers), nano graphite (simulated by two layers of stacked graphene (GRA)), and fullerene (e.g., C_60_) on the aggregation of Aβ_42_. The distance between two stacked C_3_N/GRA was set at approximately 0.33 nm, while the distance between two C_60_ molecules was larger than 1 nm. Four peptides were randomly placed around C_3_N/GRA/C_60_ with minimum distances larger than 1.5 nm. Subsequently, the C_3_N/GRA/C_60_ + peptides complexes were solvated in a water box with dimensions of 13.0 nm × 13.0 nm × 13.0 nm. The number of water molecules in the water box was 70,911, 70,918, and 71,274, respectively, for the C_3_N + peptides, GRA + peptides, and C_60_ + peptides systems. For each of the three systems, two independent 300 ns production runs were conducted for subsequent analysis.

### MD simulations

The MD simulations were carried out using the GROMACS-4.6.6^60^ software package with AMBER99SB-ILDN force field^61^. The VMD software was adopted to visualize the trajectories and configurations of the MD simulations^62,63^. The TIP3P water model was adopted for solvent molecules^64^. Long-range electrostatic interactions were conducted with the particle mesh Ewald method^65^. The vdW interactions were calculated with a smooth cutoff distance of 1.2 nm. Each solvated system was first minimized using the conjugate gradient method and succeeded by a 10 ns NPT relaxation at 300 K and 1 bar. During production runs, the simulation temperature and pressure were fixed at 300 K and 1 bar with the v−rescale thermostat and Parrinello−Rahman coupling scheme^66,67^, respectively. A time step of 2.0 fs was used, and coordinates were collected every 20 ps. For each system, three independent 1000 ns trajectories were collected for the analysis. Periodic boundary conditions were introduced in all directions. All solute bonds were constrained at their equilibrium values by employing the LINCS algorithm^68^, and water geometry was constrained with the SETTLE algorithm^69^. Electrostatic surface potential of C_3_N was calculated using the Adaptive Poisson-Boltzmann Solver^70,71^.

### Reporting summary

Further information on research design is available in the Nature Portfolio Reporting Summary linked to this article.

### Supplementary information

Supplementary Information

Peer review file

Description of Additional Supplementary Files

Supplementary Data 1

Reporting Summary

### Source data

Source data

## Data Availability

The data that support the findings of this paper are available in the paper and supplementary information files. All the raw data are provided in a Source Data file. The PDB data-base used in the study includes PDB ID: 1Z0Q [10.2210/pdb1Z0Q/pdb]. The 3D model of C_3_N nanodot (in pdb format) constructed in this study is provided in Supplementary Data 1. Source data are provided with this paper.
