# Supplementary material for: C3N nanodots inhibits Aβ peptides aggregation pathogenic path in Alzheimer’s disease
Source: Nat Commun. 2023 Sep 15;14:5718. doi: 10.1038/s41467-023-41489-y (PMC10504243; doi:10.1038/s41467-023-41489-y)
Supplement: Supplementary file 1 — Supplementary Information [file 41467_2023_41489_MOESM1_ESM.pdf]

Supporting Information for

C_3N Nanodots Inhibits Aβ Peptides Aggregation Pathogenic Path in Alzheimer's Disease

Xiuhua Yin[1,2#], Hong Zhou[1#], Mengling Zhang [2,3#], Juan Su4, Xiao Wang [2], Sijie Li4 , Zaixing Yang[1,4*], Zhenhui Kang[2,3]*, Ruhong Zhou[1,5]*

1. Institute of Quantitative Biology, Shanghai Institute for Advanced Study, College of Life Sciences, Zhejiang University, Hangzhou 310027, China
2. Jiangsu Key Laboratory for Carbon-based Functional Materials and Devices, Institute of Functional Nano and Soft Materials (FUNSOM), Soochow University, Suzhou 215123, China
3. Macao Institute of Materials Science and Engineering (MIMSE), MUST−SUDA Joint Research Center for Advanced Functional Materials, Macau University of Science and Technology, Taipa 999078, Macao, China.
4. Key Laboratory of Radiation Medicine and Protection, School for Radiological and Interdisciplinary Sciences (RAD-X), Soochow University, Suzhou 215123, China
5. Department of Chemistry, Columbia University, New York, New York 10027, United States;

[#]These authors contribute equally.

*Corresponding authors: rhzhou@zju.edu.cn (R. Zhou); zhkang@suda.edu.cn (Z. Kang); zxyang@suda.edu.cn (Z. Yang)

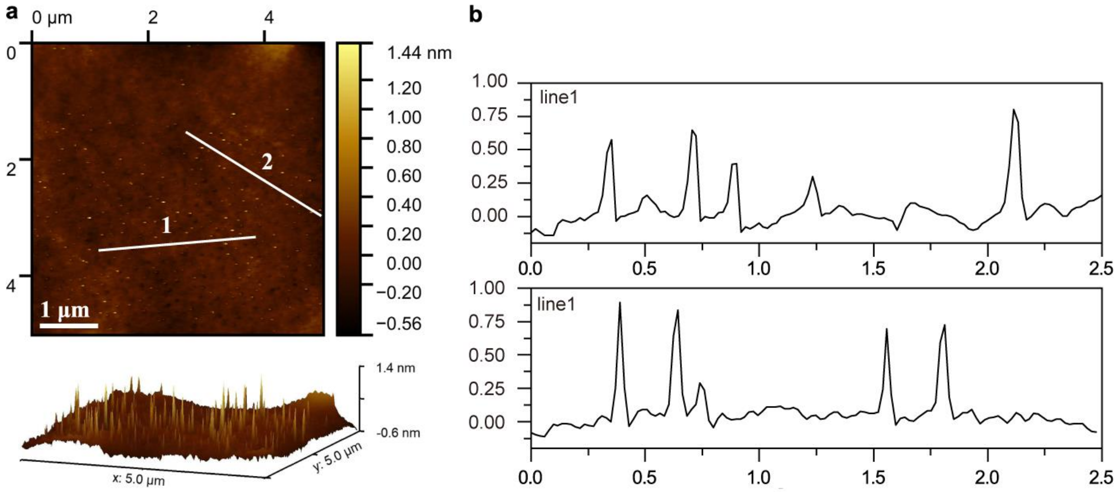

Supplementary Figure 1**.** Morphological characterization of C_3N nanodots. (a) Atomic force microscope (AFM) images of C_3N nanodots. (b) The height-profile plots corresponding to the while lines in AFM image. Scale bar = 1 µm. Source data are provided as a Source Data file.

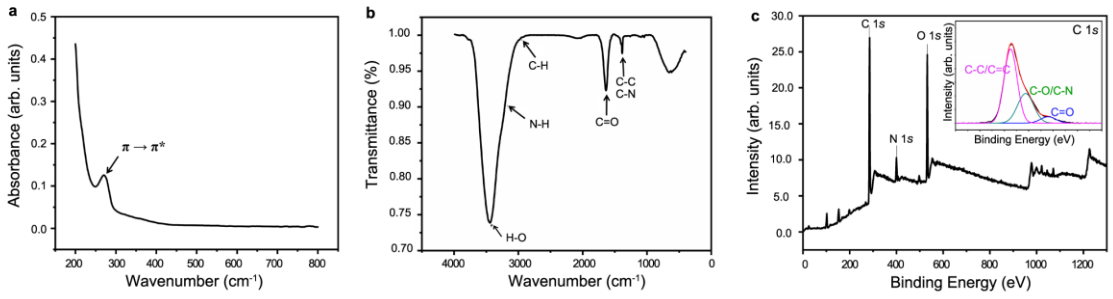

Supplementary Figure 2**.** Spectrum characterization of C_3N nanodots. (a) Ultraviolet–visible spectrum, (b) Fourier transform infrared spectrum, and (c) X-ray photoelectron spectroscopy of C_3N nanodots. arb. units = arb arbitrary units. Source data are provided as a Source Data file.

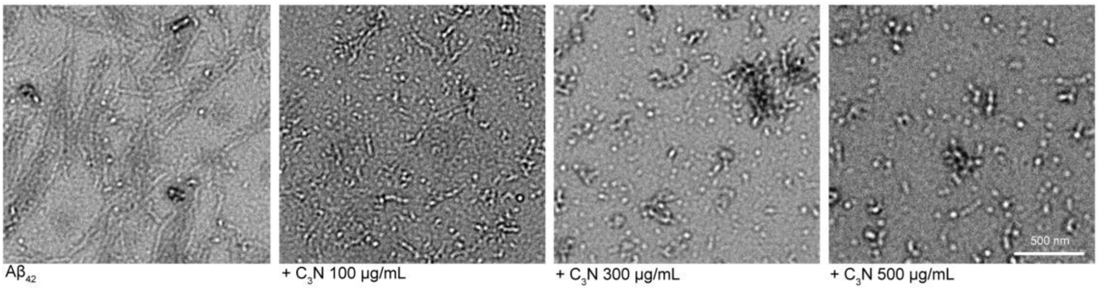

Supplementary Figure 3**.** Transmission electron microscope (TEM) images of $A\beta_{42}$ peptides treated with/without C_3N nanodots at different concentrations for 24 hours. Representative micrographs from three independent experiments with similar results.

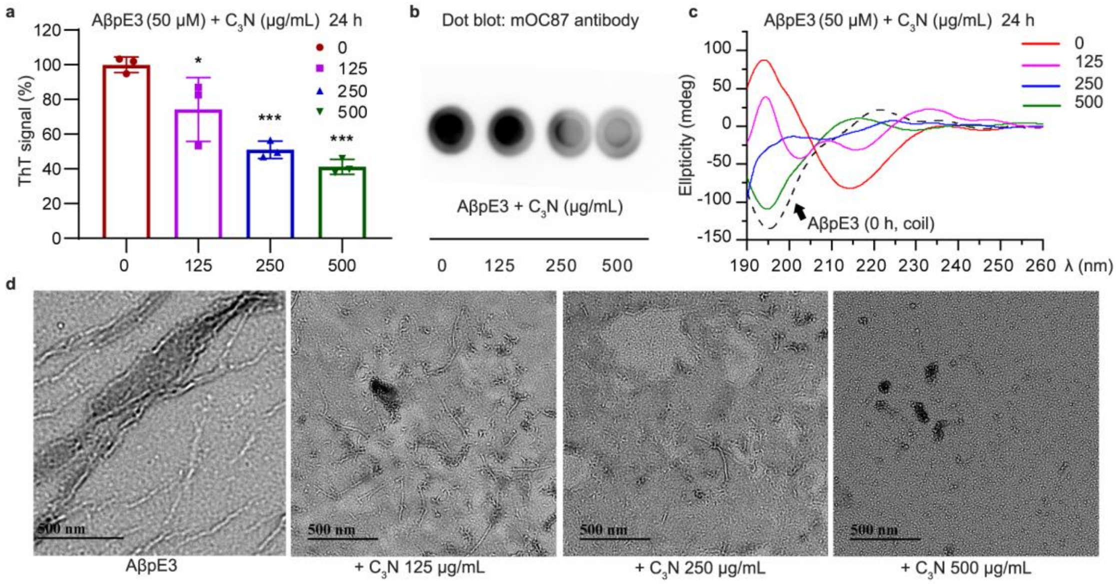

Supplementary Figure 4. C_3N nanodots inhibit N-truncated Aβ peptides (AβpE3) fibrillization in vitro. (a) Thioflavin T (ThT) fluorescence analysis of the effect of C_3N nanodots on the aggregation of AβpE3 peptides (50 μM) after 24-hour treatment with different concentrations of C_3N nanodots. Data are presented as mean ± SD and signals were normalized by setting the ThT signals of control group to 100% (n = 3). Statistical significance was determined by one-way ANOVA with $P < 0.05$ considered statistically significant (P = 0.0338, 0.0009 and 0.0003, respectively). *$P < 0.05$, ***$P < 0.001$. (b) Dot blot assay measuring the levels of amyloid fiber formation under different conditions using an Aβ fibrils conformation-specific antibody (mOC87 antibody) at time = 24 hours. (c) Circular dichroism (CD) spectra of AβpE3 peptides at 0 and 24 hours, without C_3N nanodots and after incubation with C_3N nanodots for 24 hours. (d) TEM images of AβpE3 peptides under various conditions after 24-hour incubation. Representative micrographs in (b and d) are from three independent experiments with similar results. Source data are provided as a Source Data file.

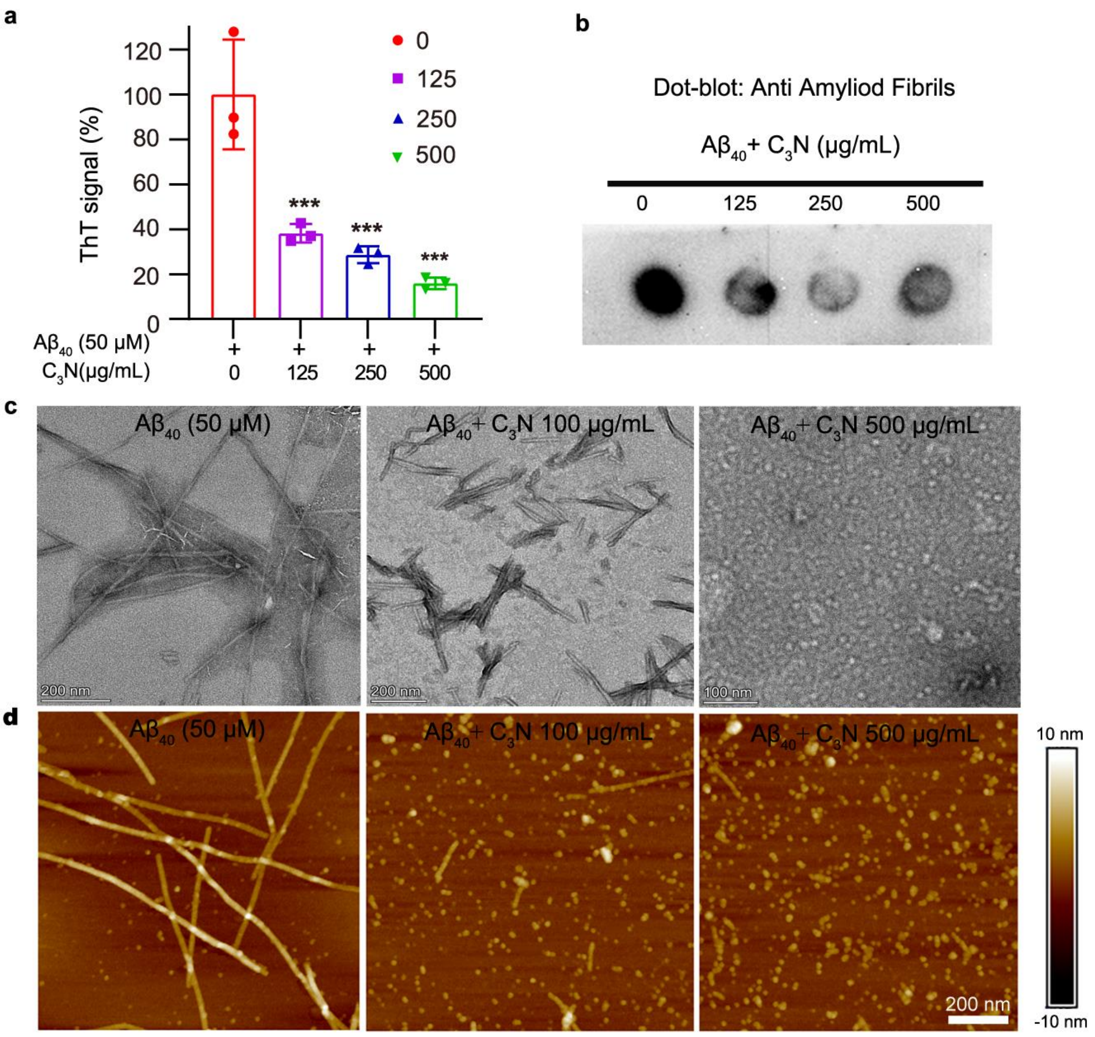

Supplementary Figure 5. C_3N nanodots inhibit $A\beta_{40}$ peptides aggregation in vitro. (a) ThT fluorescence analysis of the effect of C_3N nanodots on the aggregation of $A\beta_{40}$ peptides (50 μM) after 24-hours treatment with different concentrations of C_3N nanodots. Data are presented as mean ± SD and signals were normalized by setting the ThT signals of control group to 100%. n = 3 for each group (P = 0.0008, 0.0003 and 0.0001, respectively), ***$P < 0.001$ as determined by one-way ANOVA. (b) Dot blot assay measuring the levels of amyloid fiber formation under different conditions using mOC87 antibody, at time = 24 hours. (c) TEM and (d) AFM images of $A\beta_{40}$ peptides under various conditions after 24-hour incubation. Representative micrographs in (b-d) from three independent experiments with similar results. Source data are provided as a Source Data file.

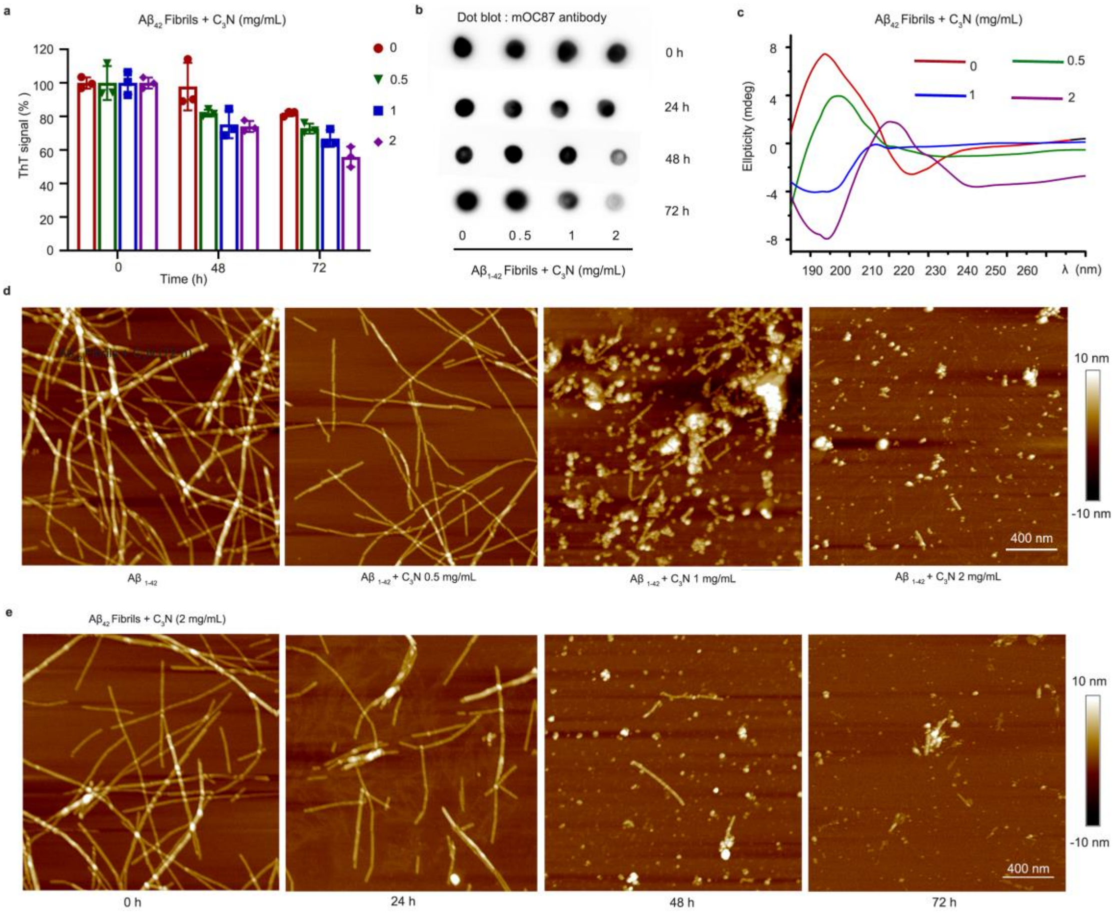

Supplementary Figure 6. C_3N nanodots disaggregate the preformed $A\beta_{42}$ mature fibrils in vitro. (a) ThT fluorescence analysis of the impact of C_3N nanodots on the $A\beta_{42}$ mature fibrils after 0-, 48- and 72-hour treatment with different concentrations of C_3N nanodots. *n* = 3 for each group and signals were normalized by setting the ThT signals of control group to 100%. (b) Dot blot assay measuring the residual levels of preformed $A\beta_{42}$ mature fibrils after treatment with different concentrations of C_3N nanodots for 0-, 24-, 48- and 72-hour using mOC87 antibody. (c) CD spectra of $A\beta_{42}$ mature fibrils treatment with different concentrations of C_3N nanodots for 72 hours. (e) AFM images of $A\beta_{42}$ mature fibrils treatment with different concentrations of C_3N nanodots for 72 hours. (f) TEM images of $A\beta_{42}$ mature fibrils after treatment with C_3N nanodots at 2 mg/mL for 0-, 24-, 48- and 72-hour. Source data are provided as a Source Data file.

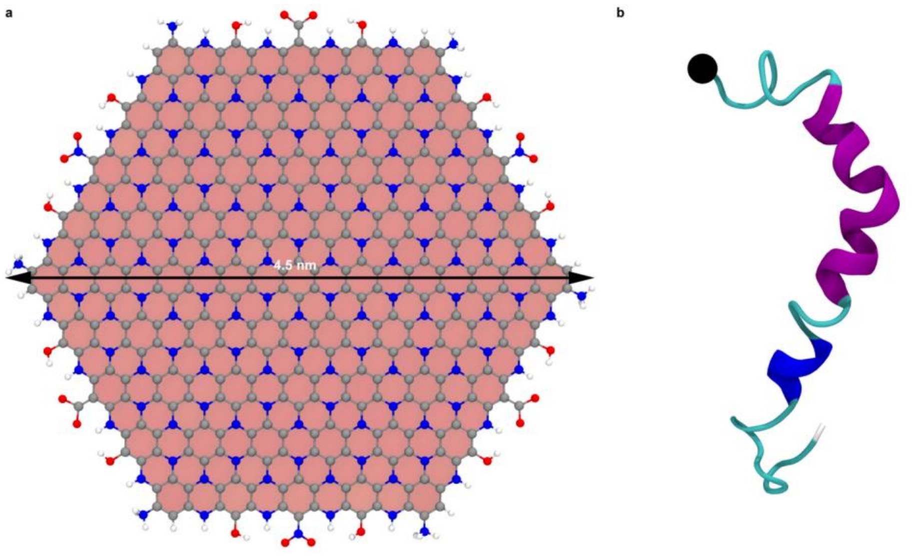

Supplementary Figure 7**.** Simulation models of C_3N nanodot and monomeric Aβ_{42} peptide. (a) C_3N model and (b) the initial Aβ_{42} structure (PDB ID: 1Z0Q) used in the molecular dynamics simulations.

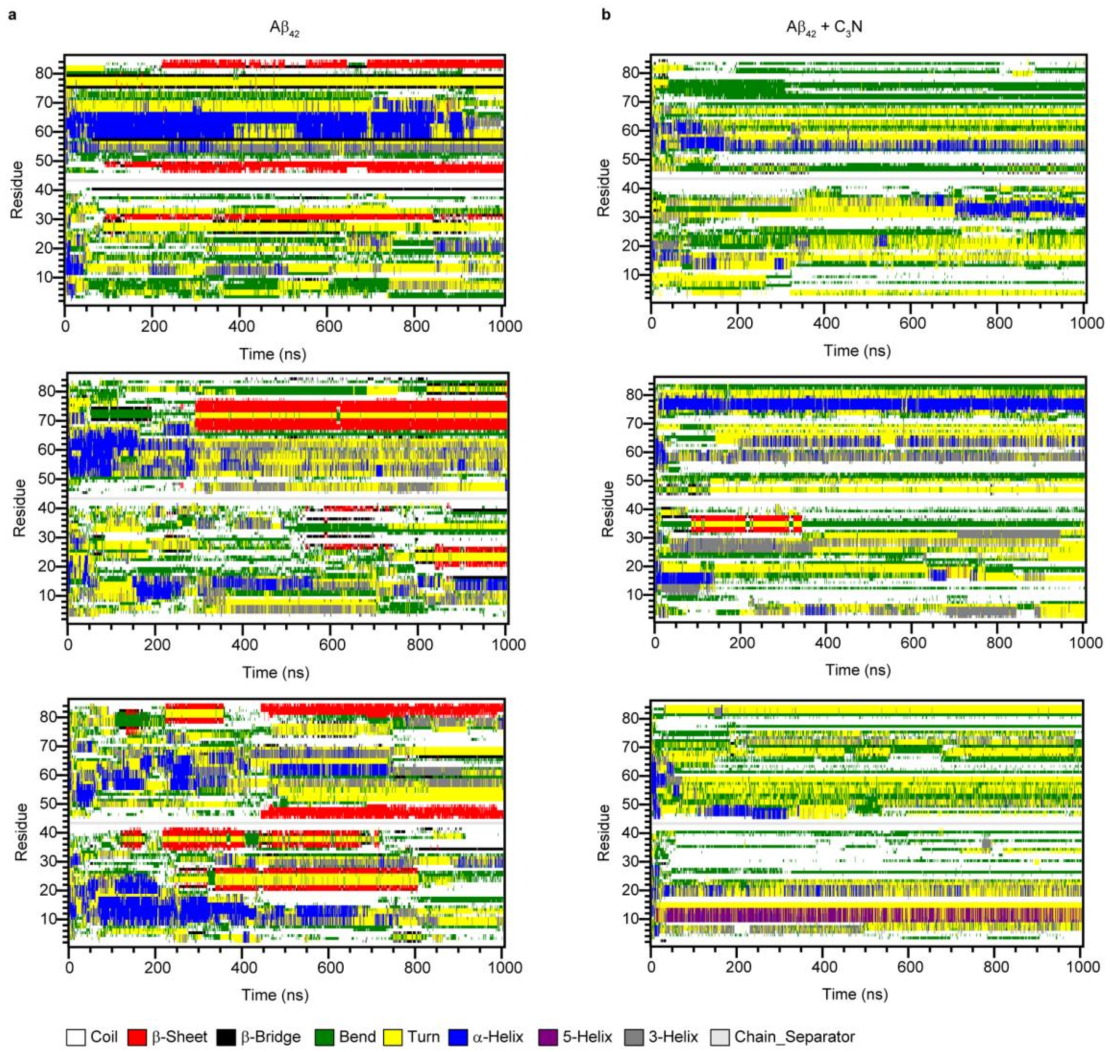

Supplementary Figure 8**.** The influence of C_3N nanodot on the secondary structure of $A\beta_{42}$ peptides. Time evolutions of the secondary structure of $A\beta_{42}$ peptides (a) in the absence or (b) in the presence of C_3N nanodot. Three panels from top to bottom in each figure represent three independent runs of each system. Here, the secondary structures of each residue were assigned according to the DSSP definition. Source data are provided as a Source Data file.

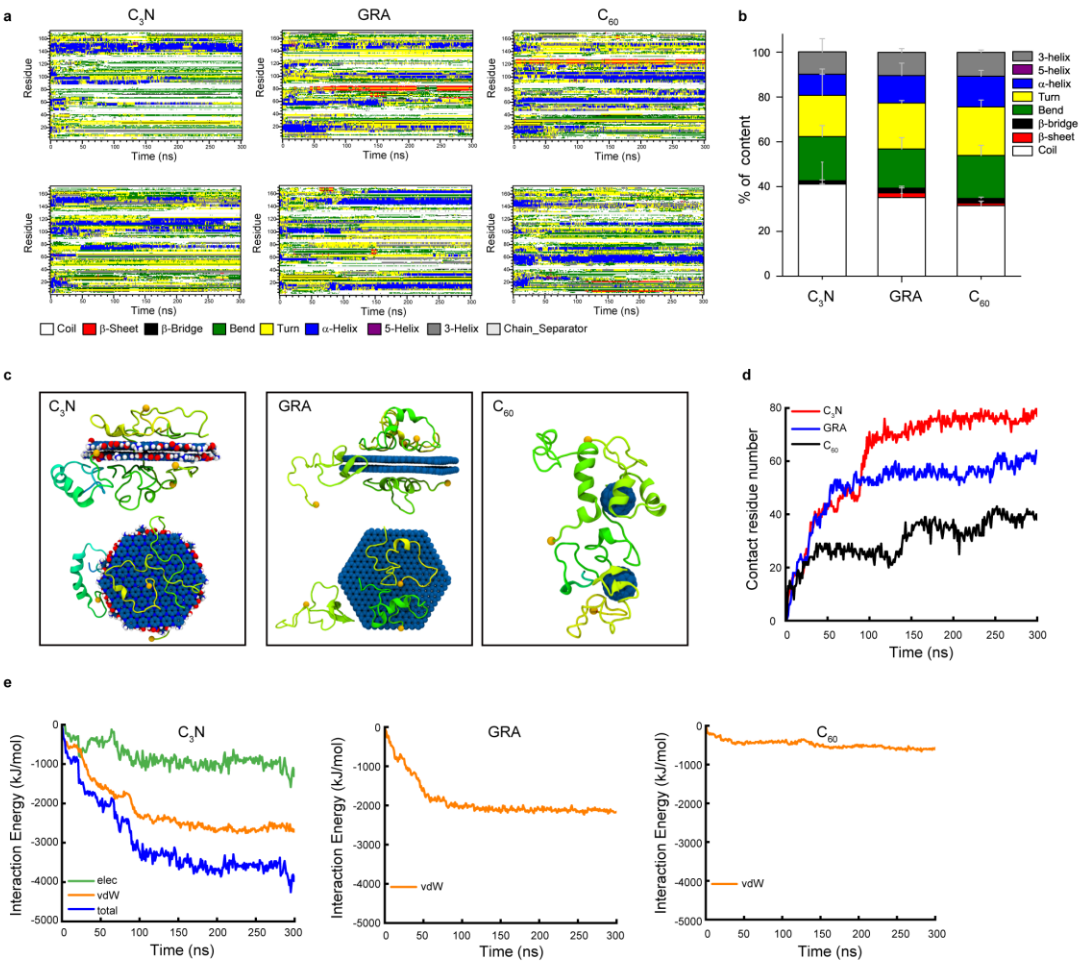

Supplementary Figure 9**.** Comparison of the influence of stacked C_3N nanodots (two layers), nano graphite (stacked graphene (GRA)) and fullerenes on the aggregation of Aβ42 peptides. (a) Time evolutions of the secondary structure of each residue in two Aβ42 peptides in the presence of C_3N-nanodots, stacked GRA and C_{60} molecules, respectively. (b) The proportions of each structural component in the peptides. The secondary structures of residues were assigned using the DSSP definition. (c) The final binding configurations. (d) Residual contact number between Aβ42 and three nanodots. (e) The nonbonded interaction energies (including electrostatic (elec), van der Waals (vdW) interactions, and a total of them) between C_3N nanodots, stacked GRA and C_{60} molecules and peptides during the process. Source data are provided as a Source Data file.

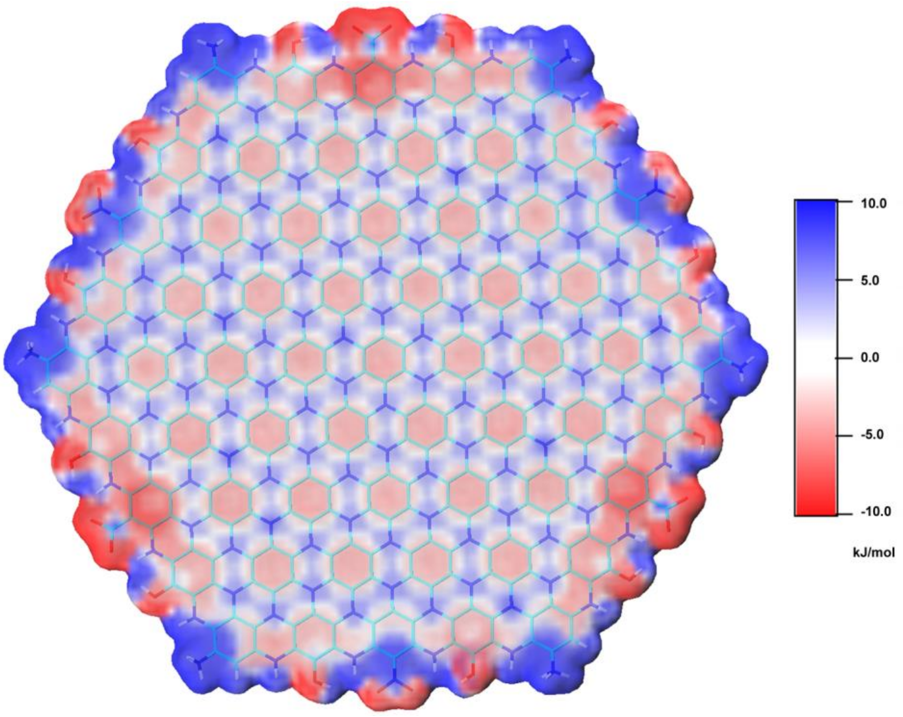

Supplementary Figure 10. Electrostatic surface potentials of C_3N. Negative, neutral, and positive potentials are displayed in red, white and blue, respectively.

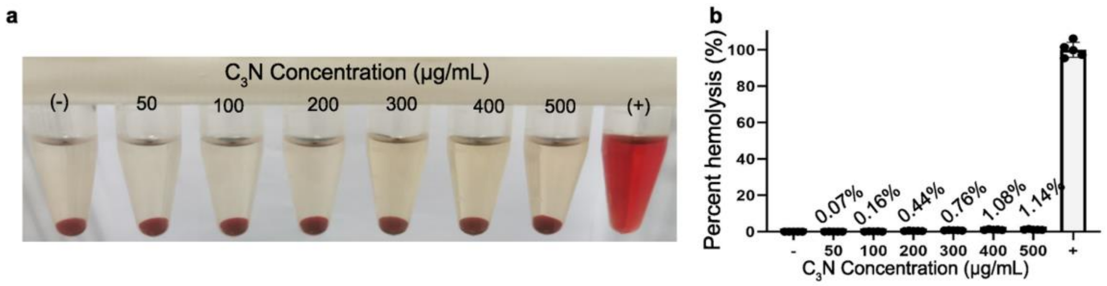

Supplementary Figure 11**.** Hemolysis activity of C_3N nanodots. (a) The hemolysis activity of C_3N nanodots via direct observation of color changes of the RBC centrifuge supernatant after incubation with C_3N nanodots at different concentrations (50–500 μg/mL) under 37°C for 3 hours. (+) and (−) symbols represented the positive and negative control, respectively. The images are from one experiment representative of five independent experiments with similar results. (b) Hemolysis ratio was calculated by spectrophotometric analysis of RBC centrifuge supernatant. Data are shown as the mean ± SD (*n* = 5). of three independent experiments. Source data are provided as a Source Data file.

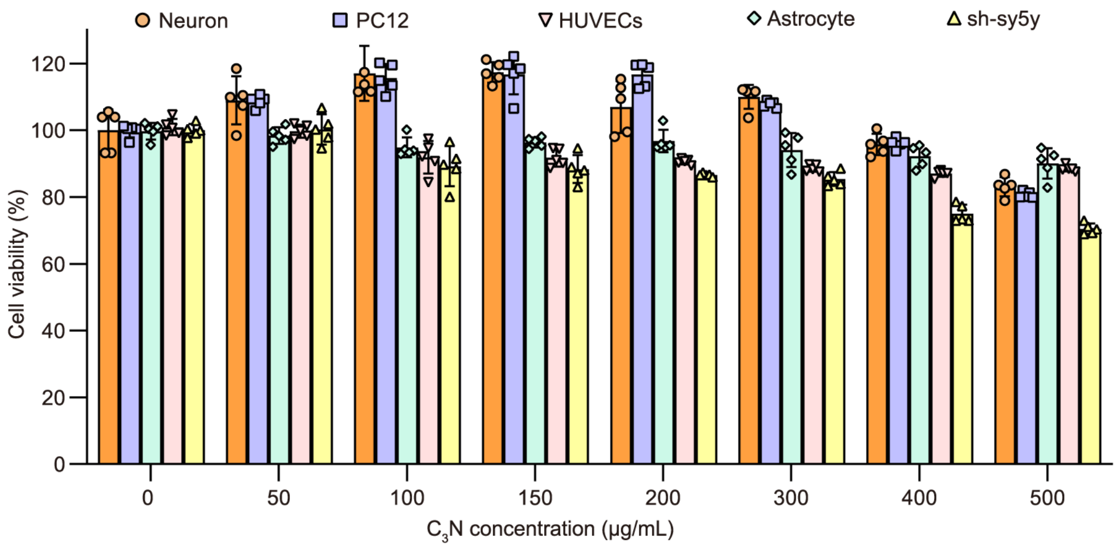

Supplementary Figure 12**.** Cytotoxicity of C_3N nanodots in different cell lines. Cells are incubated with varying concentrations of C_3N nanodots for 24 hours and subsequently assessed using a CCK-8 kit. *n* = 5 per group, data are shown as the mean ± SD. Source data are provided as a Source Data file.

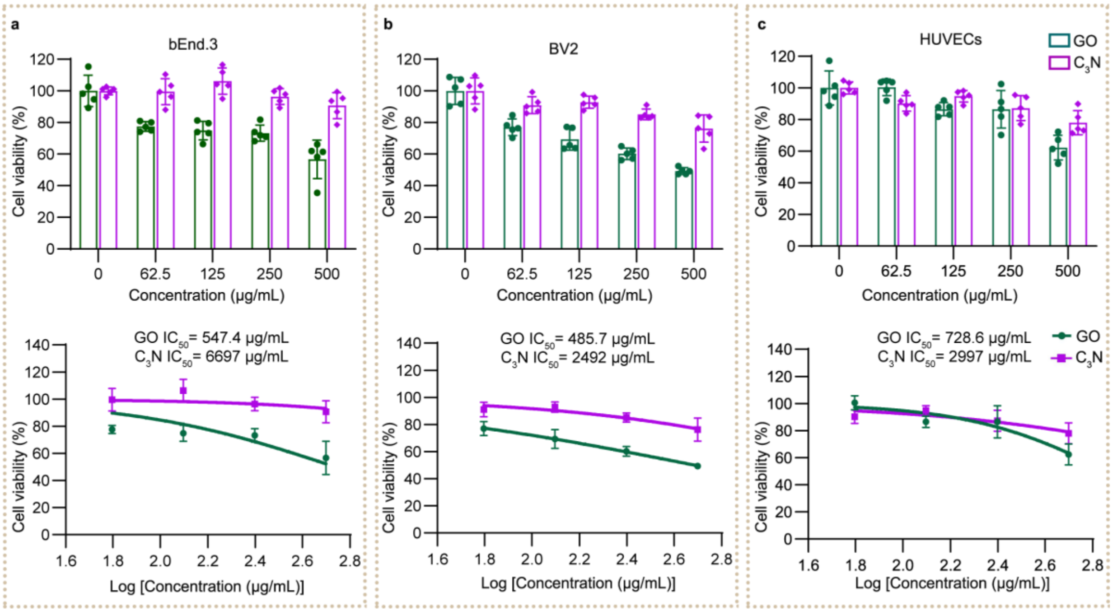

Supplementary Figure 13. Comparison of cytocompatibility between C_3N nanodots and graphene oxide (GO) nanosheets. Comparative assessment of cytocompatibility between C_3N nanodots and GO nanosheets in bEnd.3, BV2, and HUVECs cell lines. The top panel displays cell viability measured by CCK8 assay, while the bottom panel presents the IC_{50} values for the respective cell lines. *n* = 5 per group, data are shown as the mean ± SD. Source data are provided as a Source Data file.

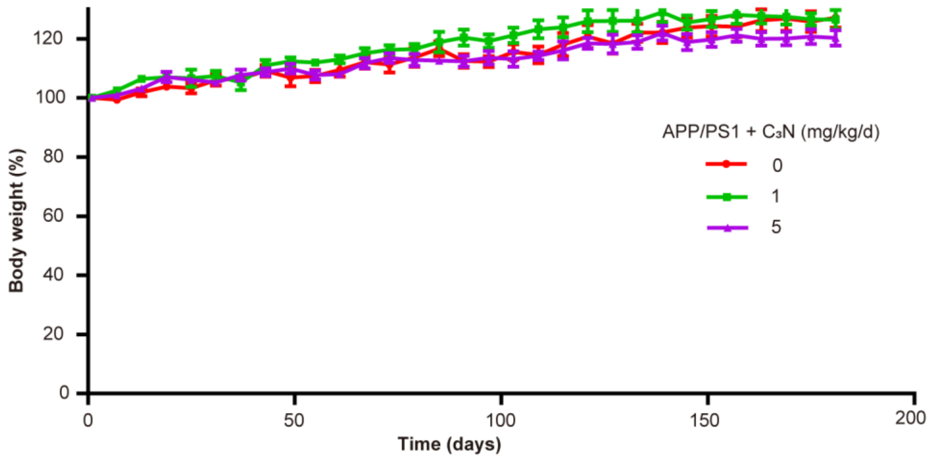

Supplementary Figure 14**.** Monitoring changes in body weight of mice in each group during the entire C_3N nanodots administration period. Data are shown as the mean ± SD (*n* = 6 mice per group). Source data are provided as a Source Data file.

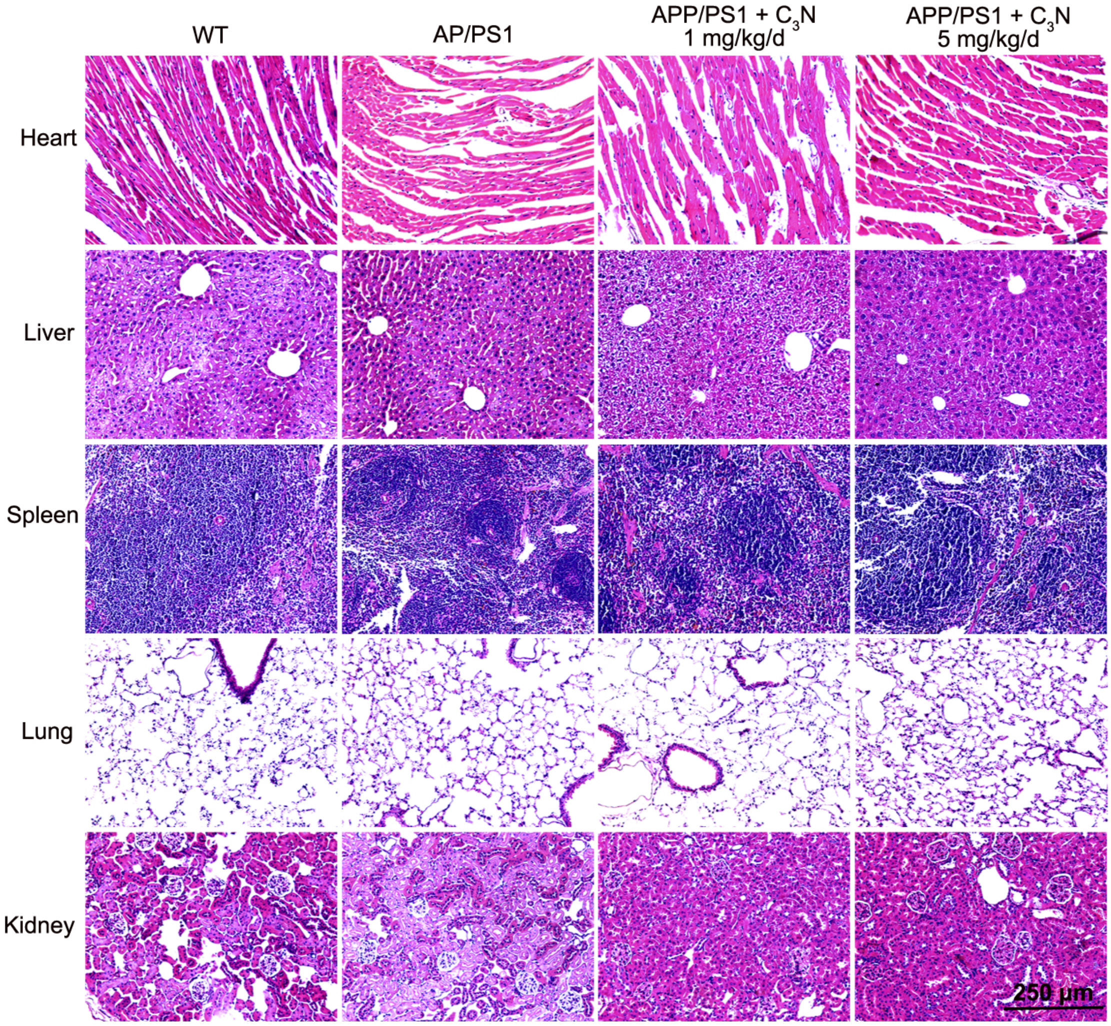

Supplementary Figure 15**.** Histopathological (H&E) analyses of important organs of mice in each group. The images are representative of three independent mice per group.

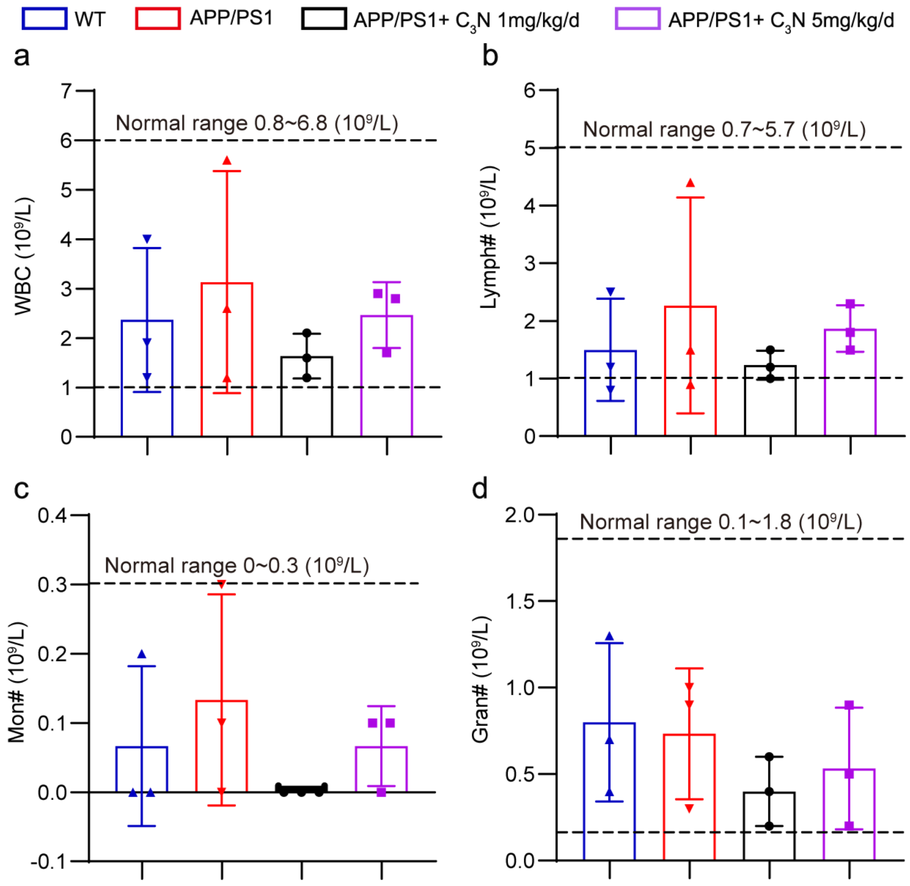

Supplementary Figure 16. The effect of C_3N nanodots on the inflammation of mice. The inflammation indexes of mice after treatment with C_3N nanodots for six months at the dose of 1 mg/kg/d and 5 mg/kg/d. (a) White blood cell count (WBC); (b) Lymphocyte count (Lymph#); (c) Monocyte count (Mon#); and (d) Granulocyte count (Gran#). *n* = 3 mice per group, data are shown as the mean ± SD. Source data are provided as a Source Data file.

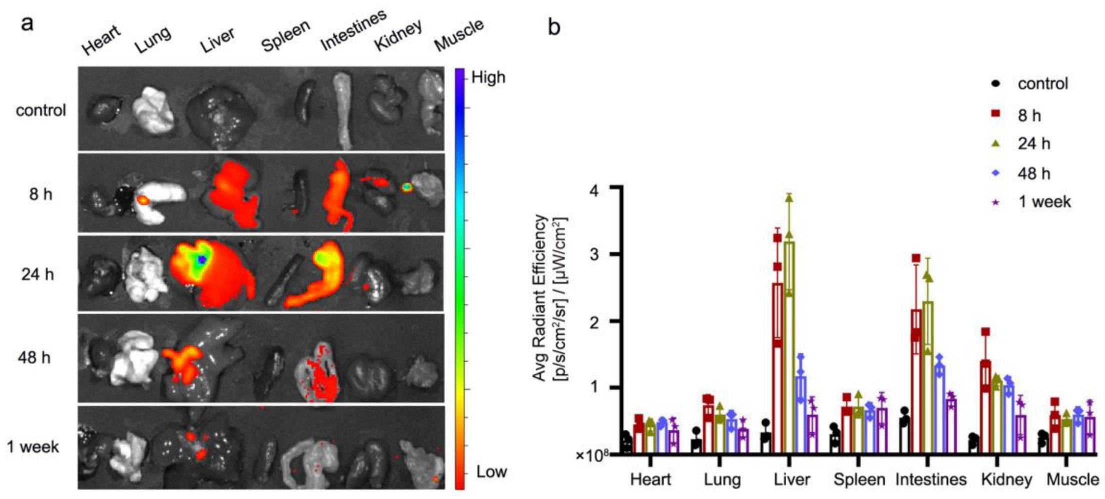

Supplementary Figure 17. Biodistribution of C_3N nanodots in key organs of mice. (a and b) Biodistribution of Cy5.5-modified (denoted as C_3N-Cy5.5) C_3N nanodots after intraperitoneal injection (i.p.) at the dose of 200 mg/kg at different time points. *n* = 3 mice per group, data are shown as the mean ± SD. Increasing fluorescence intensity is illustrated using colors ranging from red to blue. Source data are provided as a Source Data file.

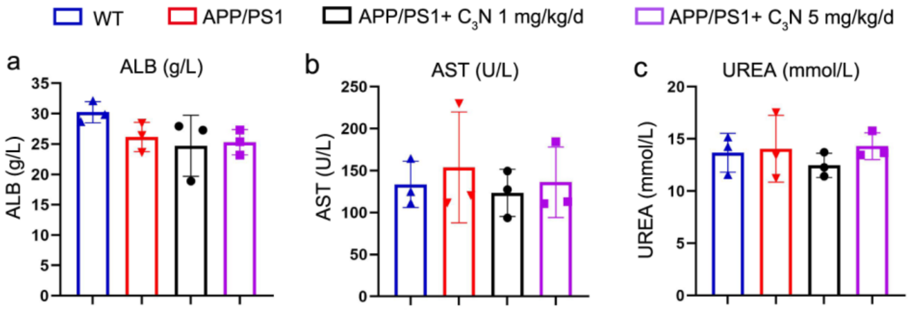

Supplementary Figure 18**.** The influence of C_3N nanodots on the liver and kidney functions in mice. The following liver and kidney function indicators of mice after treatment with C_3N nanodots at the dose of 1 mg/kg/d and 5 mg/kg/d for six months were measured. (a) Aspartate aminotransferase (AST); (b) Albumin (ALB); and (c) Urea (UREA). *n* = 3 mice per group, data are shown as the mean ± SD. Source data are provided as a Source Data file.

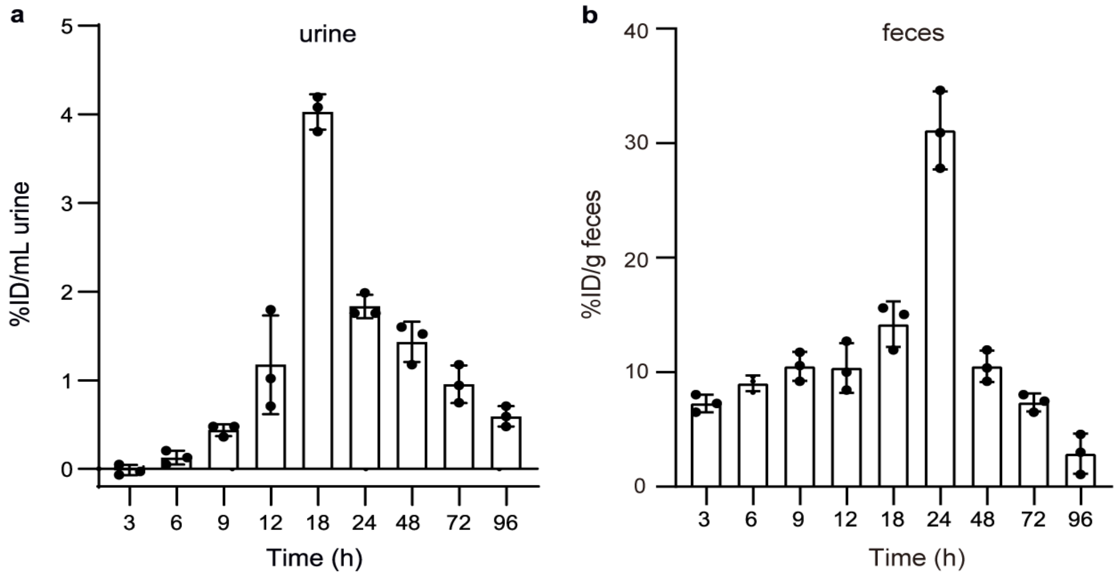

Supplementary Figure 19. Routes of excretion for C_3N nanodots. Excretion of C_3N-Cy5.5 nanodots from mice at various time points following i.p. administration at a dose of 100 mg/kg via (a) urination and (b) defecation pathways. Error bars represent the standard deviation of the excretion data obtained from three mice per group. *n* = 3 mice per group, data are shown as the mean ± SD. Source data are provided as a Source Data file.

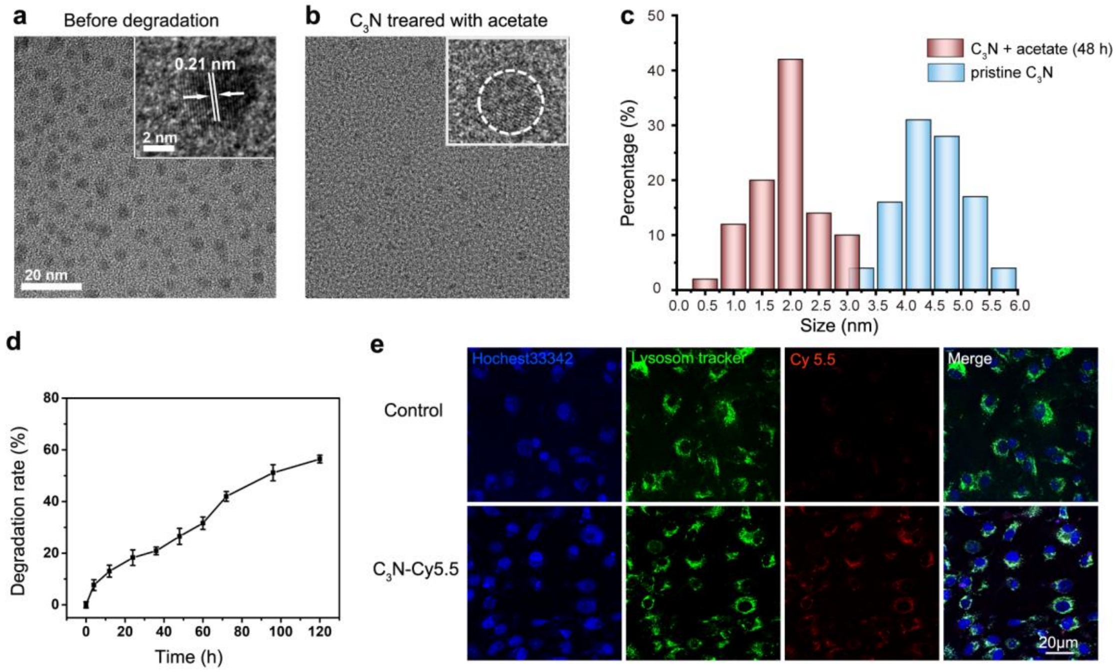

Supplementary Figure 20. Degradation of nanodots under acidic condition similar to lysosomes. (a and b) TEM images (top right corner, high resolution TEM image) and (c) the lateral size distribution of C_3N nanodots before/after incubated with 0.3 M acetate buffer at pH = 5.0 for 12 hours. (d) The time evolution of degradation rate of C_3N nanodots as measured by UV-vis spectra. (e) Confocal microscopic images of bEnd.3 cells after a 12-hour incubation with 2 mg/mL Cy5.5-labelled C_3N nanodots. C_3N-Cy5.5 nanodots exhibit a red color due to Cy5.5 (ex/em: 673/692 nm). The intracellular localization of C_3N nanodots (red) can be clearly observed, co-located with LysoTracker Green (green), while Hochest33342 (blue) was used for staining cell nuclei (scale bar = 20 μm). Source data are provided as a Source Data file.

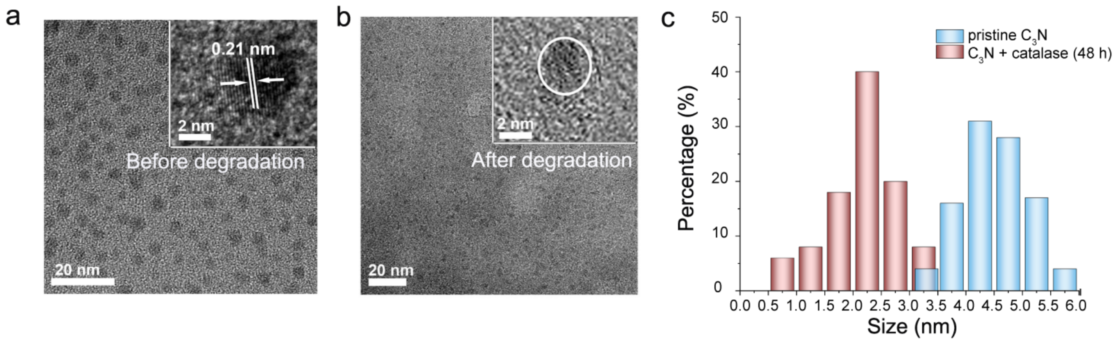

Supplementary Figure 21**.** Degradation of C_3N nanodots in the presence of catalase with physiological concentrations of H_2O_2. (a and b) TEM images (top right corner, high resolution TEM image) and (c) the lateral size distribution of C_3N nanodots in the presence/absence of catalase and the physiological concentration (100 μM) of H_2O_2 at 37 °C for one week. Source data are provided as a Source Data file.
