# Supplementary material for: C3N nanodots inhibits Aβ peptides aggregation pathogenic path in Alzheimer’s disease
Source: Nat Commun. 2023 Sep 15;14:5718. doi: 10.1038/s41467-023-41489-y (PMC10504243; doi:10.1038/s41467-023-41489-y)
Supplement: Supplementary file 3 — Description of Additional Supplementary Files [file 41467_2023_41489_MOESM3_ESM.pdf]

<u>Description of Additional Supplementary File</u>

Supplementary Data 1: The 3D model of C3N nanodot (in pdb format) constructed in this study.
