# Supplementary material for: C3N nanodots inhibits Aβ peptides aggregation pathogenic path in Alzheimer’s disease
Source: Nat Commun. 2023 Sep 15;14:5718. doi: 10.1038/s41467-023-41489-y (PMC10504243; doi:10.1038/s41467-023-41489-y)
Supplement: Supplementary file 4 — Supplementary Data 1 [file 41467_2023_41489_MOESM4_ESM.pdf]

```
CRYST1   80.000   80.000   80.000  90.00  90.00  90.00 P 1           1
ATOM      1  C1  C3N X   1      33.910  22.450  39.960  0.00  0.00
ATOM      2  C2  C3N X   1      31.470  22.450  39.970  0.00  0.00
ATOM      3  C3  C3N X   1      38.760  22.430  40.090  0.00  0.00
ATOM      4  C4  C3N X   1      36.320  22.450  40.060  0.00  0.00
ATOM      5  C5  C3N X   1      43.640  22.480  40.040  0.00  0.00
ATOM      6  C6  C3N X   1      41.200  22.430  40.020  0.00  0.00
ATOM      7  C7  C3N X   1      48.480  22.440  40.030  0.00  0.00
ATOM      8  C8  C3N X   1      46.050  22.470  40.080  0.00  0.00
ATOM      9  C9  C3N X   1      31.480  26.670  40.010  0.00  0.00
ATOM     10  C10 C3N X   1      29.040  26.660  40.020  0.00  0.00
ATOM     11  C11 C3N X   1      30.250  24.560  40.050  0.00  0.00
ATOM     12  C12 C3N X   1      36.350  26.670  40.020  0.00  0.00
ATOM     13  C13 C3N X   1      33.920  26.670  40.010  0.00  0.00
ATOM     14  C14 C3N X   1      35.130  24.560  40.020  0.00  0.00
ATOM     15  C15 C3N X   1      41.210  26.670  39.990  0.00  0.00
ATOM     16  C16 C3N X   1      38.780  26.670  39.990  0.00  0.00
ATOM     17  C17 C3N X   1      39.990  24.550  40.000  0.00  0.00
ATOM     18  C18 C3N X   1      46.070  26.680  40.020  0.00  0.00
ATOM     19  C19 C3N X   1      43.640  26.680  40.000  0.00  0.00
ATOM     20  C20 C3N X   1      44.850  24.580  40.010  0.00  0.00
ATOM     21  C21 C3N X   1      50.950  26.660  40.000  0.00  0.00
ATOM     22  C22 C3N X   1      48.510  26.670  40.020  0.00  0.00
ATOM     23  C23 C3N X   1      49.720  24.550  40.030  0.00  0.00
ATOM     24  C24 C3N X   1      29.050  30.870  40.000  0.00  0.00
ATOM     25  C25 C3N X   1      26.600  30.870  40.000  0.00  0.00
ATOM     26  C26 C3N X   1      27.830  28.760  40.000  0.00  0.00
ATOM     27  C27 C3N X   1      33.930  30.880  40.080  0.00  0.00
ATOM     28  C28 C3N X   1      31.490  30.880  40.040  0.00  0.00
ATOM     29  C29 C3N X   1      32.710  28.780  40.040  0.00  0.00
ATOM     30  C30 C3N X   1      38.790  30.880  39.990  0.00  0.00
ATOM     31  C31 C3N X   1      36.360  30.880  40.020  0.00  0.00
ATOM     32  C32 C3N X   1      37.570  28.780  40.020  0.00  0.00
ATOM     33  C33 C3N X   1      43.640  30.890  40.000  0.00  0.00
ATOM     34  C34 C3N X   1      41.210  30.880  39.980  0.00  0.00
ATOM     35  C35 C3N X   1      42.430  28.780  40.000  0.00  0.00
ATOM     36  C36 C3N X   1      48.500  30.880  39.980  0.00  0.00
ATOM     37  C37 C3N X   1      46.070  30.890  40.000  0.00  0.00
ATOM     38  C38 C3N X   1      47.290  28.780  39.990  0.00  0.00
ATOM     39  C39 C3N X   1      53.390  30.880  39.990  0.00  0.00
ATOM     40  C40 C3N X   1      50.940  30.880  39.950  0.00  0.00
ATOM     41  C41 C3N X   1      52.160  28.770  39.980  0.00  0.00
ATOM     42  C42 C3N X   1      26.640  35.080  40.000  0.00  0.00
ATOM     43  C43 C3N X   1      24.210  35.080  39.970  0.00  0.00
ATOM     44  C44 C3N X   1      25.400  32.960  40.000  0.00  0.00
ATOM     45  C45 C3N X   1      31.500  35.090  40.080  0.00  0.00
ATOM     46  C46 C3N X   1      29.070  35.090  40.060  0.00  0.00
ATOM     47  C47 C3N X   1      30.280  32.980  40.060  0.00  0.00
ATOM     48  C48 C3N X   1      36.360  35.090  39.970  0.00  0.00
ATOM     49  C49 C3N X   1      33.930  35.090  40.000  0.00  0.00
ATOM     50  C50 C3N X   1      35.150  32.990  40.010  0.00  0.00
ATOM     51  C51 C3N X   1      41.210  35.090  39.990  0.00  0.00
ATOM     52  C52 C3N X   1      38.790  35.090  40.000  0.00  0.00
ATOM     53  C53 C3N X   1      40.000  32.990  39.940  0.00  0.00
ATOM     54  C54 C3N X   1      46.070  35.090  40.020  0.00  0.00
ATOM     55  C55 C3N X   1      43.640  35.090  39.990  0.00  0.00
ATOM     56  C56 C3N X   1      44.860  32.990  40.000  0.00  0.00
```

ATOM	57	C57	C3N	X	1	50.930	35.090	40.060	0.00	0.00
ATOM	58	C58	C3N	X	1	48.500	35.090	40.080	0.00	0.00
ATOM	59	C59	C3N	X	1	49.720	32.990	40.050	0.00	0.00
ATOM	60	C60	C3N	X	1	55.800	35.100	39.980	0.00	0.00
ATOM	61	C61	C3N	X	1	53.370	35.090	39.990	0.00	0.00
ATOM	62	C62	C3N	X	1	54.590	32.980	39.980	0.00	0.00
ATOM	63	C63	C3N	X	1	24.220	39.300	39.900	0.00	0.00
ATOM	64	C64	C3N	X	1	21.790	39.280	39.940	0.00	0.00
ATOM	65	C65	C3N	X	1	23.010	37.180	39.920	0.00	0.00
ATOM	66	C66	C3N	X	1	29.080	39.300	40.000	0.00	0.00
ATOM	67	C67	C3N	X	1	26.650	39.300	39.970	0.00	0.00
ATOM	68	C68	C3N	X	1	27.860	37.190	40.000	0.00	0.00
ATOM	69	C69	C3N	X	1	33.930	39.300	39.950	0.00	0.00
ATOM	70	C70	C3N	X	1	31.500	39.300	39.990	0.00	0.00
ATOM	71	C71	C3N	X	1	32.720	37.200	40.000	0.00	0.00
ATOM	72	C72	C3N	X	1	38.790	39.300	40.040	0.00	0.00
ATOM	73	C73	C3N	X	1	36.360	39.300	39.950	0.00	0.00
ATOM	74	C74	C3N	X	1	37.580	37.200	40.000	0.00	0.00
ATOM	75	C75	C3N	X	1	43.640	39.300	39.970	0.00	0.00
ATOM	76	C76	C3N	X	1	41.220	39.300	39.970	0.00	0.00
ATOM	77	C77	C3N	X	1	42.430	37.200	40.000	0.00	0.00
ATOM	78	C78	C3N	X	1	48.500	39.300	40.000	0.00	0.00
ATOM	79	C79	C3N	X	1	46.070	39.300	40.000	0.00	0.00
ATOM	80	C80	C3N	X	1	47.290	37.200	40.010	0.00	0.00
ATOM	81	C81	C3N	X	1	53.370	39.300	40.010	0.00	0.00
ATOM	82	C82	C3N	X	1	50.930	39.300	39.990	0.00	0.00
ATOM	83	C83	C3N	X	1	52.150	37.200	39.990	0.00	0.00
ATOM	84	C84	C3N	X	1	58.270	39.290	40.040	0.00	0.00
ATOM	85	C85	C3N	X	1	55.810	39.300	40.070	0.00	0.00
ATOM	86	C86	C3N	X	1	57.030	37.200	40.030	0.00	0.00
ATOM	87	C87	C3N	X	1	21.780	43.500	39.940	0.00	0.00
ATOM	88	C88	C3N	X	1	20.570	41.390	39.980	0.00	0.00
ATOM	89	C89	C3N	X	1	26.640	43.510	40.030	0.00	0.00
ATOM	90	C90	C3N	X	1	24.210	43.510	39.970	0.00	0.00
ATOM	91	C91	C3N	X	1	25.430	41.400	39.960	0.00	0.00
ATOM	92	C92	C3N	X	1	31.500	43.510	40.020	0.00	0.00
ATOM	93	C93	C3N	X	1	29.070	43.510	40.070	0.00	0.00
ATOM	94	C94	C3N	X	1	30.290	41.410	40.030	0.00	0.00
ATOM	95	C95	C3N	X	1	36.360	43.510	40.020	0.00	0.00
ATOM	96	C96	C3N	X	1	33.930	43.510	40.010	0.00	0.00
ATOM	97	C97	C3N	X	1	35.150	41.400	40.000	0.00	0.00
ATOM	98	C98	C3N	X	1	41.220	43.510	39.950	0.00	0.00
ATOM	99	C99	C3N	X	1	38.790	43.510	39.980	0.00	0.00
ATOM	100	C100	C3N	X	1	40.000	41.400	39.990	0.00	0.00
ATOM	101	C101	C3N	X	1	46.070	43.510	40.040	0.00	0.00
ATOM	102	C102	C3N	X	1	43.640	43.510	39.980	0.00	0.00
ATOM	103	C103	C3N	X	1	44.860	41.400	39.990	0.00	0.00
ATOM	104	C104	C3N	X	1	50.940	43.510	40.010	0.00	0.00
ATOM	105	C105	C3N	X	1	48.500	43.510	40.050	0.00	0.00
ATOM	106	C106	C3N	X	1	49.720	41.410	40.010	0.00	0.00
ATOM	107	C107	C3N	X	1	55.810	43.510	40.000	0.00	0.00
ATOM	108	C108	C3N	X	1	53.370	43.510	40.010	0.00	0.00
ATOM	109	C109	C3N	X	1	54.590	41.410	40.050	0.00	0.00
ATOM	110	C110	C3N	X	1	58.260	43.510	39.920	0.00	0.00
ATOM	111	C111	C3N	X	1	59.490	41.410	39.990	0.00	0.00
ATOM	112	C112	C3N	X	1	24.170	47.730	39.950	0.00	0.00
ATOM	113	C113	C3N	X	1	22.990	45.600	39.970	0.00	0.00
ATOM	114	C114	C3N	X	1	29.060	47.720	40.000	0.00	0.00

ATOM	115	C115	C3N	X	1	26.620	47.730	40.010	0.00	0.00
ATOM	116	C116	C3N	X	1	27.850	45.620	40.030	0.00	0.00
ATOM	117	C117	C3N	X	1	33.930	47.710	39.990	0.00	0.00
ATOM	118	C118	C3N	X	1	31.500	47.720	39.960	0.00	0.00
ATOM	119	C119	C3N	X	1	32.710	45.610	39.990	0.00	0.00
ATOM	120	C120	C3N	X	1	38.790	47.710	40.010	0.00	0.00
ATOM	121	C121	C3N	X	1	36.360	47.710	40.030	0.00	0.00
ATOM	122	C122	C3N	X	1	37.570	45.610	40.000	0.00	0.00
ATOM	123	C123	C3N	X	1	43.640	47.710	40.010	0.00	0.00
ATOM	124	C124	C3N	X	1	41.220	47.710	40.000	0.00	0.00
ATOM	125	C125	C3N	X	1	42.430	45.610	39.990	0.00	0.00
ATOM	126	C126	C3N	X	1	48.510	47.710	39.990	0.00	0.00
ATOM	127	C127	C3N	X	1	46.070	47.710	40.010	0.00	0.00
ATOM	128	C128	C3N	X	1	47.290	45.610	40.020	0.00	0.00
ATOM	129	C129	C3N	X	1	53.390	47.730	40.010	0.00	0.00
ATOM	130	C130	C3N	X	1	50.950	47.720	39.950	0.00	0.00
ATOM	131	C131	C3N	X	1	52.160	45.620	40.000	0.00	0.00
ATOM	132	C132	C3N	X	1	55.840	47.730	40.040	0.00	0.00
ATOM	133	C133	C3N	X	1	57.030	45.600	39.970	0.00	0.00
ATOM	134	C134	C3N	X	1	26.660	51.940	39.940	0.00	0.00
ATOM	135	C135	C3N	X	1	25.400	49.850	39.940	0.00	0.00
ATOM	136	C136	C3N	X	1	31.500	51.930	40.050	0.00	0.00
ATOM	137	C137	C3N	X	1	29.080	51.930	40.040	0.00	0.00
ATOM	138	C138	C3N	X	1	30.280	49.820	39.990	0.00	0.00
ATOM	139	C139	C3N	X	1	36.360	51.920	39.980	0.00	0.00
ATOM	140	C140	C3N	X	1	33.930	51.920	40.000	0.00	0.00
ATOM	141	C141	C3N	X	1	35.140	49.820	40.000	0.00	0.00
ATOM	142	C142	C3N	X	1	41.220	51.930	39.970	0.00	0.00
ATOM	143	C143	C3N	X	1	38.790	51.930	39.960	0.00	0.00
ATOM	144	C144	C3N	X	1	40.000	49.820	39.990	0.00	0.00
ATOM	145	C145	C3N	X	1	46.070	51.930	40.070	0.00	0.00
ATOM	146	C146	C3N	X	1	43.640	51.930	40.030	0.00	0.00
ATOM	147	C147	C3N	X	1	44.860	49.820	40.050	0.00	0.00
ATOM	148	C148	C3N	X	1	50.940	51.920	39.980	0.00	0.00
ATOM	149	C149	C3N	X	1	48.500	51.920	40.020	0.00	0.00
ATOM	150	C150	C3N	X	1	49.720	49.820	39.960	0.00	0.00
ATOM	151	C151	C3N	X	1	53.370	51.940	40.020	0.00	0.00
ATOM	152	C152	C3N	X	1	54.620	49.840	40.030	0.00	0.00
ATOM	153	C153	C3N	X	1	29.060	56.150	40.000	0.00	0.00
ATOM	154	C154	C3N	X	1	27.860	54.040	39.970	0.00	0.00
ATOM	155	C155	C3N	X	1	33.930	56.140	39.980	0.00	0.00
ATOM	156	C156	C3N	X	1	31.500	56.160	40.010	0.00	0.00
ATOM	157	C157	C3N	X	1	32.710	54.040	40.030	0.00	0.00
ATOM	158	C158	C3N	X	1	38.790	56.160	40.040	0.00	0.00
ATOM	159	C159	C3N	X	1	36.350	56.140	40.020	0.00	0.00
ATOM	160	C160	C3N	X	1	37.570	54.040	40.000	0.00	0.00
ATOM	161	C161	C3N	X	1	43.650	56.150	39.910	0.00	0.00
ATOM	162	C162	C3N	X	1	41.210	56.160	39.950	0.00	0.00
ATOM	163	C163	C3N	X	1	42.430	54.040	39.960	0.00	0.00
ATOM	164	C164	C3N	X	1	48.500	56.140	40.000	0.00	0.00
ATOM	165	C165	C3N	X	1	46.070	56.160	39.990	0.00	0.00
ATOM	166	C166	C3N	X	1	47.290	54.040	40.010	0.00	0.00
ATOM	167	C167	C3N	X	1	50.950	56.130	39.970	0.00	0.00
ATOM	168	C168	C3N	X	1	52.160	54.020	40.000	0.00	0.00
ATOM	169	C169	C3N	X	1	29.020	23.850	40.080	0.00	0.00
ATOM	170	C170	C3N	X	1	27.820	25.960	40.030	0.00	0.00
ATOM	171	C171	C3N	X	1	31.470	23.860	40.010	0.00	0.00
ATOM	172	C172	C3N	X	1	33.910	23.860	40.000	0.00	0.00

```
ATOM    173 C173 C3N X   1       32.700  25.970  40.000  0.00  0.00
ATOM    174 C174 C3N X   1       36.340  23.860  40.040  0.00  0.00
ATOM    175 C175 C3N X   1       38.780  23.840  40.040  0.00  0.00
ATOM    176 C176 C3N X   1       37.560  25.960  40.000  0.00  0.00
ATOM    177 C177 C3N X   1       41.200  23.850  40.010  0.00  0.00
ATOM    178 C178 C3N X   1       43.640  23.880  39.990  0.00  0.00
ATOM    179 C179 C3N X   1       42.420  25.970  39.990  0.00  0.00
ATOM    180 C180 C3N X   1       46.070  23.880  40.040  0.00  0.00
ATOM    181 C181 C3N X   1       48.500  23.850  40.070  0.00  0.00
ATOM    182 C182 C3N X   1       47.290  25.970  40.040  0.00  0.00
ATOM    183 C183 C3N X   1       50.940  23.840  39.980  0.00  0.00
ATOM    184 C184 C3N X   1       52.170  25.950  39.990  0.00  0.00
ATOM    185 C185 C3N X   1       26.610  28.050  40.000  0.00  0.00
ATOM    186 C186 C3N X   1       25.370  30.160  40.000  0.00  0.00
ATOM    187 C187 C3N X   1       29.050  28.070  40.000  0.00  0.00
ATOM    188 C188 C3N X   1       31.480  28.070  40.010  0.00  0.00
ATOM    189 C189 C3N X   1       30.270  30.180  40.010  0.00  0.00
ATOM    190 C190 C3N X   1       33.920  28.080  40.030  0.00  0.00
ATOM    191 C191 C3N X   1       36.350  28.080  40.040  0.00  0.00
ATOM    192 C192 C3N X   1       35.140  30.180  40.050  0.00  0.00
ATOM    193 C193 C3N X   1       38.780  28.070  40.000  0.00  0.00
ATOM    194 C194 C3N X   1       41.210  28.080  40.000  0.00  0.00
ATOM    195 C195 C3N X   1       40.000  30.180  39.990  0.00  0.00
ATOM    196 C196 C3N X   1       43.640  28.080  40.000  0.00  0.00
ATOM    197 C197 C3N X   1       46.080  28.080  40.000  0.00  0.00
ATOM    198 C198 C3N X   1       44.860  30.190  40.000  0.00  0.00
ATOM    199 C199 C3N X   1       48.510  28.070  39.990  0.00  0.00
ATOM    200 C200 C3N X   1       50.940  28.060  39.980  0.00  0.00
ATOM    201 C201 C3N X   1       49.730  30.180  39.960  0.00  0.00
ATOM    202 C202 C3N X   1       53.370  28.060  40.010  0.00  0.00
ATOM    203 C203 C3N X   1       54.620  30.170  40.010  0.00  0.00
ATOM    204 C204 C3N X   1       24.170  32.250  40.000  0.00  0.00
ATOM    205 C205 C3N X   1       22.980  34.380  40.000  0.00  0.00
ATOM    206 C206 C3N X   1       26.610  32.270  40.000  0.00  0.00
ATOM    207 C207 C3N X   1       29.050  32.280  40.020  0.00  0.00
ATOM    208 C208 C3N X   1       27.850  34.380  40.030  0.00  0.00
ATOM    209 C209 C3N X   1       31.490  32.280  40.050  0.00  0.00
ATOM    210 C210 C3N X   1       33.920  32.280  40.050  0.00  0.00
ATOM    211 C211 C3N X   1       32.710  34.390  40.050  0.00  0.00
ATOM    212 C212 C3N X   1       36.350  32.290  40.000  0.00  0.00
ATOM    213 C213 C3N X   1       38.780  32.290  39.960  0.00  0.00
ATOM    214 C214 C3N X   1       37.570  34.390  39.990  0.00  0.00
ATOM    215 C215 C3N X   1       41.210  32.290  39.960  0.00  0.00
ATOM    216 C216 C3N X   1       43.640  32.290  40.000  0.00  0.00
ATOM    217 C217 C3N X   1       42.430  34.390  39.990  0.00  0.00
ATOM    218 C218 C3N X   1       46.080  32.290  40.000  0.00  0.00
ATOM    219 C219 C3N X   1       48.510  32.280  40.020  0.00  0.00
ATOM    220 C220 C3N X   1       47.290  34.390  40.050  0.00  0.00
ATOM    221 C221 C3N X   1       50.940  32.280  40.000  0.00  0.00
ATOM    222 C222 C3N X   1       53.380  32.280  39.990  0.00  0.00
ATOM    223 C223 C3N X   1       52.150  34.380  40.020  0.00  0.00
ATOM    224 C224 C3N X   1       55.820  32.270  39.980  0.00  0.00
ATOM    225 C225 C3N X   1       57.020  34.410  39.990  0.00  0.00
ATOM    226 C226 C3N X   1       21.800  36.470  39.960  0.00  0.00
ATOM    227 C227 C3N X   1       20.590  38.550  39.970  0.00  0.00
ATOM    228 C228 C3N X   1       24.220  36.480  39.940  0.00  0.00
ATOM    229 C229 C3N X   1       26.640  36.480  39.990  0.00  0.00
ATOM    230 C230 C3N X   1       25.430  38.590  39.940  0.00  0.00
```

ATOM	231	C231	C3N	X	1	29.070	36.490	40.030	0.00	0.00
ATOM	232	C232	C3N	X	1	31.500	36.490	40.030	0.00	0.00
ATOM	233	C233	C3N	X	1	30.290	38.590	40.000	0.00	0.00
ATOM	234	C234	C3N	X	1	33.930	36.490	39.980	0.00	0.00
ATOM	235	C235	C3N	X	1	36.360	36.490	39.960	0.00	0.00
ATOM	236	C236	C3N	X	1	35.140	38.600	39.920	0.00	0.00
ATOM	237	C237	C3N	X	1	38.790	36.490	40.030	0.00	0.00
ATOM	238	C238	C3N	X	1	41.210	36.490	40.010	0.00	0.00
ATOM	239	C239	C3N	X	1	40.000	38.600	40.020	0.00	0.00
ATOM	240	C240	C3N	X	1	43.640	36.490	40.000	0.00	0.00
ATOM	241	C241	C3N	X	1	46.070	36.490	40.010	0.00	0.00
ATOM	242	C242	C3N	X	1	44.860	38.600	39.990	0.00	0.00
ATOM	243	C243	C3N	X	1	48.500	36.490	40.040	0.00	0.00
ATOM	244	C244	C3N	X	1	50.940	36.490	40.020	0.00	0.00
ATOM	245	C245	C3N	X	1	49.720	38.600	39.990	0.00	0.00
ATOM	246	C246	C3N	X	1	53.370	36.490	39.980	0.00	0.00
ATOM	247	C247	C3N	X	1	55.800	36.500	40.000	0.00	0.00
ATOM	248	C248	C3N	X	1	54.590	38.600	40.020	0.00	0.00
ATOM	249	C249	C3N	X	1	58.240	36.490	40.020	0.00	0.00
ATOM	250	C250	C3N	X	1	59.500	38.580	39.980	0.00	0.00
ATOM	251	C251	C3N	X	1	21.780	40.690	39.930	0.00	0.00
ATOM	252	C252	C3N	X	1	24.220	40.700	39.910	0.00	0.00
ATOM	253	C253	C3N	X	1	23.000	42.800	39.920	0.00	0.00
ATOM	254	C254	C3N	X	1	26.640	40.700	39.990	0.00	0.00
ATOM	255	C255	C3N	X	1	29.070	40.700	40.020	0.00	0.00
ATOM	256	C256	C3N	X	1	27.860	42.810	40.050	0.00	0.00
ATOM	257	C257	C3N	X	1	31.500	40.700	40.010	0.00	0.00
ATOM	258	C258	C3N	X	1	33.930	40.700	39.980	0.00	0.00
ATOM	259	C259	C3N	X	1	32.720	42.800	40.010	0.00	0.00
ATOM	260	C260	C3N	X	1	36.360	40.700	40.010	0.00	0.00
ATOM	261	C261	C3N	X	1	38.790	40.700	40.070	0.00	0.00
ATOM	262	C262	C3N	X	1	37.570	42.800	40.020	0.00	0.00
ATOM	263	C263	C3N	X	1	41.220	40.700	39.930	0.00	0.00
ATOM	264	C264	C3N	X	1	43.640	40.700	39.950	0.00	0.00
ATOM	265	C265	C3N	X	1	42.430	42.800	39.930	0.00	0.00
ATOM	266	C266	C3N	X	1	46.070	40.700	40.010	0.00	0.00
ATOM	267	C267	C3N	X	1	48.500	40.700	40.020	0.00	0.00
ATOM	268	C268	C3N	X	1	47.290	42.800	40.070	0.00	0.00
ATOM	269	C269	C3N	X	1	50.930	40.700	40.000	0.00	0.00
ATOM	270	C270	C3N	X	1	53.370	40.700	40.020	0.00	0.00
ATOM	271	C271	C3N	X	1	52.150	42.810	40.010	0.00	0.00
ATOM	272	C272	C3N	X	1	55.810	40.700	40.090	0.00	0.00
ATOM	273	C273	C3N	X	1	58.270	40.700	40.050	0.00	0.00
ATOM	274	C274	C3N	X	1	57.040	42.810	39.990	0.00	0.00
ATOM	275	C275	C3N	X	1	24.210	44.910	40.000	0.00	0.00
ATOM	276	C276	C3N	X	1	26.640	44.910	40.030	0.00	0.00
ATOM	277	C277	C3N	X	1	25.400	47.030	40.000	0.00	0.00
ATOM	278	C278	C3N	X	1	29.070	44.910	40.040	0.00	0.00
ATOM	279	C279	C3N	X	1	31.500	44.910	40.010	0.00	0.00
ATOM	280	C280	C3N	X	1	30.280	47.010	39.990	0.00	0.00
ATOM	281	C281	C3N	X	1	33.930	44.910	40.010	0.00	0.00
ATOM	282	C282	C3N	X	1	36.360	44.910	40.010	0.00	0.00
ATOM	283	C283	C3N	X	1	35.140	47.010	40.020	0.00	0.00
ATOM	284	C284	C3N	X	1	38.790	44.910	39.980	0.00	0.00
ATOM	285	C285	C3N	X	1	41.210	44.910	39.980	0.00	0.00
ATOM	286	C286	C3N	X	1	40.000	47.010	40.000	0.00	0.00
ATOM	287	C287	C3N	X	1	43.640	44.910	40.000	0.00	0.00
ATOM	288	C288	C3N	X	1	46.070	44.910	40.030	0.00	0.00

ATOM	289	C289	C3N	X	1	44.860	47.010	40.010	0.00	0.00
ATOM	290	C290	C3N	X	1	48.500	44.910	40.020	0.00	0.00
ATOM	291	C291	C3N	X	1	50.940	44.910	40.000	0.00	0.00
ATOM	292	C292	C3N	X	1	49.720	47.010	39.980	0.00	0.00
ATOM	293	C293	C3N	X	1	53.370	44.910	40.010	0.00	0.00
ATOM	294	C294	C3N	X	1	55.810	44.910	40.000	0.00	0.00
ATOM	295	C295	C3N	X	1	54.610	47.030	40.040	0.00	0.00
ATOM	296	C296	C3N	X	1	26.620	49.130	39.990	0.00	0.00
ATOM	297	C297	C3N	X	1	29.070	49.120	40.000	0.00	0.00
ATOM	298	C298	C3N	X	1	27.860	51.230	40.000	0.00	0.00
ATOM	299	C299	C3N	X	1	31.500	49.120	39.950	0.00	0.00
ATOM	300	C300	C3N	X	1	33.930	49.120	39.980	0.00	0.00
ATOM	301	C301	C3N	X	1	32.720	51.230	40.000	0.00	0.00
ATOM	302	C302	C3N	X	1	36.360	49.120	40.010	0.00	0.00
ATOM	303	C303	C3N	X	1	38.790	49.120	40.000	0.00	0.00
ATOM	304	C304	C3N	X	1	37.570	51.230	39.980	0.00	0.00
ATOM	305	C305	C3N	X	1	41.210	49.120	40.000	0.00	0.00
ATOM	306	C306	C3N	X	1	43.640	49.120	40.020	0.00	0.00
ATOM	307	C307	C3N	X	1	42.430	51.230	40.000	0.00	0.00
ATOM	308	C308	C3N	X	1	46.070	49.120	40.030	0.00	0.00
ATOM	309	C309	C3N	X	1	48.500	49.120	39.990	0.00	0.00
ATOM	310	C310	C3N	X	1	47.290	51.230	40.050	0.00	0.00
ATOM	311	C311	C3N	X	1	50.940	49.120	39.930	0.00	0.00
ATOM	312	C312	C3N	X	1	53.390	49.130	39.990	0.00	0.00
ATOM	313	C313	C3N	X	1	52.150	51.230	39.980	0.00	0.00
ATOM	314	C314	C3N	X	1	29.080	53.340	40.040	0.00	0.00
ATOM	315	C315	C3N	X	1	31.500	53.340	40.080	0.00	0.00
ATOM	316	C316	C3N	X	1	30.290	55.450	40.020	0.00	0.00
ATOM	317	C317	C3N	X	1	33.930	53.330	40.000	0.00	0.00
ATOM	318	C318	C3N	X	1	36.360	53.330	39.980	0.00	0.00
ATOM	319	C319	C3N	X	1	35.140	55.440	39.980	0.00	0.00
ATOM	320	C320	C3N	X	1	38.790	53.340	39.980	0.00	0.00
ATOM	321	C321	C3N	X	1	41.220	53.340	39.970	0.00	0.00
ATOM	322	C322	C3N	X	1	40.000	55.460	40.000	0.00	0.00
ATOM	323	C323	C3N	X	1	43.640	53.340	39.990	0.00	0.00
ATOM	324	C324	C3N	X	1	46.070	53.340	40.030	0.00	0.00
ATOM	325	C325	C3N	X	1	44.860	55.450	39.960	0.00	0.00
ATOM	326	C326	C3N	X	1	48.500	53.330	40.010	0.00	0.00
ATOM	327	C327	C3N	X	1	50.940	53.330	39.990	0.00	0.00
ATOM	328	C328	C3N	X	1	49.720	55.440	39.990	0.00	0.00
ATOM	329	C329	C3N	X	1	31.500	57.580	40.000	0.00	0.00
ATOM	330	C330	C3N	X	1	33.930	57.550	39.980	0.00	0.00
ATOM	331	C331	C3N	X	1	36.350	57.550	40.030	0.00	0.00
ATOM	332	C332	C3N	X	1	38.790	57.570	40.060	0.00	0.00
ATOM	333	C333	C3N	X	1	41.210	57.590	39.960	0.00	0.00
ATOM	334	C334	C3N	X	1	43.660	57.570	39.930	0.00	0.00
ATOM	335	C335	C3N	X	1	46.070	57.570	39.990	0.00	0.00
ATOM	336	C336	C3N	X	1	48.500	57.550	40.000	0.00	0.00
ATOM	337	N1	C3N	X	1	30.260	25.960	40.030	0.00	0.00
ATOM	338	N2	C3N	X	1	35.130	25.970	40.010	0.00	0.00
ATOM	339	N3	C3N	X	1	39.990	25.960	39.990	0.00	0.00
ATOM	340	N4	C3N	X	1	44.860	25.980	40.010	0.00	0.00
ATOM	341	N5	C3N	X	1	49.730	25.960	40.020	0.00	0.00
ATOM	342	N6	C3N	X	1	27.820	30.170	39.990	0.00	0.00
ATOM	343	N7	C3N	X	1	32.700	30.180	40.080	0.00	0.00
ATOM	344	N8	C3N	X	1	37.570	30.180	40.010	0.00	0.00
ATOM	345	N9	C3N	X	1	42.430	30.180	40.000	0.00	0.00
ATOM	346	N10	C3N	X	1	47.290	30.180	39.990	0.00	0.00

ATOM	347	N11	C3N	X	1	52.170	30.170	39.960	0.00	0.00
ATOM	348	N12	C3N	X	1	25.410	34.370	39.980	0.00	0.00
ATOM	349	N13	C3N	X	1	30.280	34.380	40.090	0.00	0.00
ATOM	350	N14	C3N	X	1	35.140	34.390	39.980	0.00	0.00
ATOM	351	N15	C3N	X	1	40.000	34.390	39.970	0.00	0.00
ATOM	352	N16	C3N	X	1	44.860	34.390	39.990	0.00	0.00
ATOM	353	N17	C3N	X	1	49.720	34.390	40.090	0.00	0.00
ATOM	354	N18	C3N	X	1	54.590	34.390	39.980	0.00	0.00
ATOM	355	N19	C3N	X	1	23.010	38.580	39.900	0.00	0.00
ATOM	356	N20	C3N	X	1	27.860	38.590	39.990	0.00	0.00
ATOM	357	N21	C3N	X	1	32.710	38.590	39.970	0.00	0.00
ATOM	358	N22	C3N	X	1	37.570	38.600	39.990	0.00	0.00
ATOM	359	N23	C3N	X	1	42.430	38.600	39.980	0.00	0.00
ATOM	360	N24	C3N	X	1	47.290	38.600	39.990	0.00	0.00
ATOM	361	N25	C3N	X	1	52.150	38.600	39.990	0.00	0.00
ATOM	362	N26	C3N	X	1	57.040	38.600	40.050	0.00	0.00
ATOM	363	N27	C3N	X	1	25.430	42.800	39.990	0.00	0.00
ATOM	364	N28	C3N	X	1	30.290	42.800	40.060	0.00	0.00
ATOM	365	N29	C3N	X	1	35.150	42.800	40.020	0.00	0.00
ATOM	366	N30	C3N	X	1	40.000	42.800	39.970	0.00	0.00
ATOM	367	N31	C3N	X	1	44.860	42.800	40.000	0.00	0.00
ATOM	368	N32	C3N	X	1	49.720	42.800	40.030	0.00	0.00
ATOM	369	N33	C3N	X	1	54.590	42.800	40.030	0.00	0.00
ATOM	370	N34	C3N	X	1	27.850	47.020	40.020	0.00	0.00
ATOM	371	N35	C3N	X	1	32.720	47.010	39.980	0.00	0.00
ATOM	372	N36	C3N	X	1	37.570	47.010	40.010	0.00	0.00
ATOM	373	N37	C3N	X	1	42.430	47.010	40.000	0.00	0.00
ATOM	374	N38	C3N	X	1	47.290	47.010	40.000	0.00	0.00
ATOM	375	N39	C3N	X	1	52.160	47.020	39.980	0.00	0.00
ATOM	376	N40	C3N	X	1	30.290	51.230	40.030	0.00	0.00
ATOM	377	N41	C3N	X	1	35.150	51.220	39.990	0.00	0.00
ATOM	378	N42	C3N	X	1	40.000	51.230	39.970	0.00	0.00
ATOM	379	N43	C3N	X	1	44.850	51.230	40.080	0.00	0.00
ATOM	380	N44	C3N	X	1	49.720	51.220	39.980	0.00	0.00
ATOM	381	N45	C3N	X	1	32.710	55.450	40.000	0.00	0.00
ATOM	382	N46	C3N	X	1	37.580	55.450	40.040	0.00	0.00
ATOM	383	N47	C3N	X	1	42.430	55.460	39.920	0.00	0.00
ATOM	384	N48	C3N	X	1	47.290	55.450	40.000	0.00	0.00
ATOM	385	N49	C3N	X	1	32.690	24.560	40.000	0.00	0.00
ATOM	386	N50	C3N	X	1	37.560	24.550	40.020	0.00	0.00
ATOM	387	N51	C3N	X	1	42.420	24.570	39.990	0.00	0.00
ATOM	388	N52	C3N	X	1	47.290	24.570	40.080	0.00	0.00
ATOM	389	N53	C3N	X	1	30.270	28.770	40.010	0.00	0.00
ATOM	390	N54	C3N	X	1	35.140	28.780	40.050	0.00	0.00
ATOM	391	N55	C3N	X	1	40.000	28.780	40.000	0.00	0.00
ATOM	392	N56	C3N	X	1	44.860	28.780	40.000	0.00	0.00
ATOM	393	N57	C3N	X	1	49.720	28.770	39.970	0.00	0.00
ATOM	394	N58	C3N	X	1	27.840	32.980	40.010	0.00	0.00
ATOM	395	N59	C3N	X	1	32.720	32.990	40.050	0.00	0.00
ATOM	396	N60	C3N	X	1	37.570	32.990	39.980	0.00	0.00
ATOM	397	N61	C3N	X	1	42.430	32.990	39.980	0.00	0.00
ATOM	398	N62	C3N	X	1	47.290	32.990	40.020	0.00	0.00
ATOM	399	N63	C3N	X	1	52.150	32.980	40.000	0.00	0.00
ATOM	400	N64	C3N	X	1	25.440	37.190	39.940	0.00	0.00
ATOM	401	N65	C3N	X	1	30.290	37.190	40.010	0.00	0.00
ATOM	402	N66	C3N	X	1	35.150	37.200	39.930	0.00	0.00
ATOM	403	N67	C3N	X	1	40.000	37.200	40.030	0.00	0.00
ATOM	404	N68	C3N	X	1	44.860	37.200	39.990	0.00	0.00

ATOM	405	N69	C3N	X	1	49.720	37.200	40.010	0.00	0.00
ATOM	406	N70	C3N	X	1	54.580	37.200	40.000	0.00	0.00
ATOM	407	N71	C3N	X	1	23.000	41.400	39.880	0.00	0.00
ATOM	408	N72	C3N	X	1	27.860	41.410	40.030	0.00	0.00
ATOM	409	N73	C3N	X	1	32.720	41.410	40.000	0.00	0.00
ATOM	410	N74	C3N	X	1	37.570	41.410	40.070	0.00	0.00
ATOM	411	N75	C3N	X	1	42.430	41.400	39.910	0.00	0.00
ATOM	412	N76	C3N	X	1	47.290	41.400	40.040	0.00	0.00
ATOM	413	N77	C3N	X	1	52.150	41.410	40.000	0.00	0.00
ATOM	414	N78	C3N	X	1	57.040	41.410	40.070	0.00	0.00
ATOM	415	N79	C3N	X	1	25.420	45.620	40.020	0.00	0.00
ATOM	416	N80	C3N	X	1	30.280	45.610	40.010	0.00	0.00
ATOM	417	N81	C3N	X	1	35.140	45.610	40.020	0.00	0.00
ATOM	418	N82	C3N	X	1	40.000	45.610	39.990	0.00	0.00
ATOM	419	N83	C3N	X	1	44.860	45.610	40.010	0.00	0.00
ATOM	420	N84	C3N	X	1	49.720	45.610	40.000	0.00	0.00
ATOM	421	N85	C3N	X	1	54.600	45.620	40.020	0.00	0.00
ATOM	422	N86	C3N	X	1	27.850	49.830	40.000	0.00	0.00
ATOM	423	N87	C3N	X	1	32.710	49.820	39.960	0.00	0.00
ATOM	424	N88	C3N	X	1	37.570	49.820	40.000	0.00	0.00
ATOM	425	N89	C3N	X	1	42.430	49.820	40.000	0.00	0.00
ATOM	426	N90	C3N	X	1	47.290	49.820	40.020	0.00	0.00
ATOM	427	N91	C3N	X	1	52.170	49.820	39.960	0.00	0.00
ATOM	428	N92	C3N	X	1	30.290	54.040	40.070	0.00	0.00
ATOM	429	N93	C3N	X	1	35.140	54.030	39.970	0.00	0.00
ATOM	430	N94	C3N	X	1	40.000	54.040	39.990	0.00	0.00
ATOM	431	N95	C3N	X	1	44.860	54.040	39.990	0.00	0.00
ATOM	432	N96	C3N	X	1	49.720	54.030	39.990	0.00	0.00
ATOM	433	C337	C3N	X	1	29.040	22.440	40.060	0.00	0.00
ATOM	434	C338	C3N	X	1	60.730	39.300	39.930	0.00	0.00
ATOM	435	C339	C3N	X	1	30.270	58.280	40.040	0.00	0.00
ATOM	436	C340	C3N	X	1	49.690	21.740	39.930	0.00	0.00
ATOM	437	C341	C3N	X	1	19.380	40.630	40.000	0.00	0.00
ATOM	438	C342	C3N	X	1	50.920	57.530	39.930	0.00	0.00
ATOM	439	N97	C3N	X	1	30.240	59.540	40.050	0.00	0.00
ATOM	440	N98	C3N	X	1	51.960	58.160	39.920	0.00	0.00
ATOM	441	N99	C3N	X	1	61.850	38.700	39.810	0.00	0.00
ATOM	442	N100	C3N	X	1	49.700	20.500	39.800	0.00	0.00
ATOM	443	N101	C3N	X	1	27.970	21.800	40.080	0.00	0.00
ATOM	444	N102	C3N	X	1	18.330	41.170	39.880	0.00	0.00
ATOM	445	H1	C3N	X	1	30.720	59.840	39.230	0.00	0.00
ATOM	446	H2	C3N	X	1	30.780	59.870	40.830	0.00	0.00
ATOM	447	H3	C3N	X	1	29.320	59.930	40.100	0.00	0.00
ATOM	448	H4	C3N	X	1	52.560	57.870	39.180	0.00	0.00
ATOM	449	H5	C3N	X	1	52.470	57.870	40.730	0.00	0.00
ATOM	450	H6	C3N	X	1	51.820	59.150	39.930	0.00	0.00
ATOM	451	H7	C3N	X	1	61.850	38.200	38.950	0.00	0.00
ATOM	452	H8	C3N	X	1	61.950	37.920	40.420	0.00	0.00
ATOM	453	H9	C3N	X	1	62.640	39.300	39.860	0.00	0.00
ATOM	454	H10	C3N	X	1	50.600	20.160	39.610	0.00	0.00
ATOM	455	H11	C3N	X	1	49.060	20.200	39.100	0.00	0.00
ATOM	456	H12	C3N	X	1	49.320	20.120	40.650	0.00	0.00
ATOM	457	H13	C3N	X	1	28.140	20.830	40.170	0.00	0.00
ATOM	458	H14	C3N	X	1	27.500	21.980	39.210	0.00	0.00
ATOM	459	H15	C3N	X	1	27.350	22.140	40.770	0.00	0.00
ATOM	460	H16	C3N	X	1	17.570	40.540	39.770	0.00	0.00
ATOM	461	H17	C3N	X	1	18.410	41.720	39.050	0.00	0.00
ATOM	462	H18	C3N	X	1	18.230	41.760	40.690	0.00	0.00

```
ATOM    463 C343 C3N X   1      24.110  30.830  40.000  0.00  0.00
ATOM    464 C344 C3N X   1      55.880  30.850  40.000  0.00  0.00
ATOM    465 C345 C3N X   1      40.000  58.340  40.000  0.00  0.00
ATOM    466 C346 C3N X   1      39.970  59.640  39.980  0.00  0.00
ATOM    467 C347 C3N X   1      22.970  30.180  39.980  0.00  0.00
ATOM    468 C348 C3N X   1      57.020  30.230  40.000  0.00  0.00
ATOM    469  O1  C3N X   1      38.870  60.430  40.120  0.00  0.00
ATOM    470  O2  C3N X   1      41.100  60.400  39.830  0.00  0.00
ATOM    471  O3  C3N X   1      22.850  28.820  39.970  0.00  0.00
ATOM    472  O4  C3N X   1      21.730  30.750  39.910  0.00  0.00
ATOM    473  O5  C3N X   1      58.250  30.810  39.830  0.00  0.00
ATOM    474  O6  C3N X   1      57.100  28.880  40.000  0.00  0.00
ATOM    475 C349 C3N X   1      39.980  21.670  40.030  0.00  0.00
ATOM    476 C350 C3N X   1      24.130  49.180  39.920  0.00  0.00
ATOM    477 C351 C3N X   1      55.890  49.160  40.050  0.00  0.00
ATOM    478 N103 C3N X   1      22.960  49.860  39.860  0.00  0.00
ATOM    479 N104 C3N X   1      39.960  20.330  40.000  0.00  0.00
ATOM    480 N105 C3N X   1      57.050  49.830  40.070  0.00  0.00
ATOM    481  O7  C3N X   1      22.890  51.060  39.960  0.00  0.00
ATOM    482  O8  C3N X   1      21.870  49.310  39.860  0.00  0.00
ATOM    483  O9  C3N X   1      40.960  19.670  40.020  0.00  0.00
ATOM    484  O10 C3N X   1      38.940  19.640  40.000  0.00  0.00
ATOM    485  O11 C3N X   1      57.120  51.020  39.860  0.00  0.00
ATOM    486  O12 C3N X   1      58.140  49.310  39.920  0.00  0.00
ATOM    487 C352 C3N X   1      26.590  26.650  40.000  0.00  0.00
ATOM    488 C353 C3N X   1      53.390  26.650  40.010  0.00  0.00
ATOM    489 C354 C3N X   1      21.780  35.070  40.020  0.00  0.00
ATOM    490 C355 C3N X   1      58.230  35.100  40.020  0.00  0.00
ATOM    491 C356 C3N X   1      35.140  58.250  39.990  0.00  0.00
ATOM    492 C357 C3N X   1      44.870  58.280  39.980  0.00  0.00
ATOM    493 C358 C3N X   1      35.110  21.740  39.970  0.00  0.00
ATOM    494 C359 C3N X   1      44.840  21.790  40.140  0.00  0.00
ATOM    495 C360 C3N X   1      21.780  44.900  39.940  0.00  0.00
ATOM    496 C361 C3N X   1      58.250  44.920  39.910  0.00  0.00
ATOM    497 C362 C3N X   1      26.660  53.340  39.930  0.00  0.00
ATOM    498 C363 C3N X   1      53.370  53.340  40.020  0.00  0.00
ATOM    499  O13 C3N X   1      20.610  45.560  39.860  0.00  0.00
ATOM    500  O14 C3N X   1      25.520  54.040  39.850  0.00  0.00
ATOM    501  O15 C3N X   1      35.140  59.610  39.950  0.00  0.00
ATOM    502  O16 C3N X   1      44.910  59.660  40.050  0.00  0.00
ATOM    503  O17 C3N X   1      54.520  54.070  40.100  0.00  0.00
ATOM    504  O18 C3N X   1      59.430  45.630  39.920  0.00  0.00
ATOM    505  O19 C3N X   1      59.450  34.510  40.070  0.00  0.00
ATOM    506  O20 C3N X   1      54.570  25.940  40.030  0.00  0.00
ATOM    507  O21 C3N X   1      44.800  20.490  40.520  0.00  0.00
ATOM    508  O22 C3N X   1      35.070  20.370  39.870  0.00  0.00
ATOM    509  O23 C3N X   1      25.420  25.930  39.950  0.00  0.00
ATOM    510  O24 C3N X   1      20.580  34.440  40.170  0.00  0.00
ATOM    511  H19 C3N X   1      20.770  46.490  39.910  0.00  0.00
ATOM    512  H20 C3N X   1      24.800  53.410  39.910  0.00  0.00
ATOM    513  H21 C3N X   1      36.080  59.840  40.020  0.00  0.00
ATOM    514  H22 C3N X   1      44.010  60.020  40.000  0.00  0.00
ATOM    515  H23 C3N X   1      55.250  53.500  40.380  0.00  0.00
ATOM    516  H24 C3N X   1      59.250  46.560  39.850  0.00  0.00
ATOM    517  H25 C3N X   1      59.320  33.560  40.170  0.00  0.00
ATOM    518  H26 C3N X   1      55.320  26.540  39.990  0.00  0.00
ATOM    519  H27 C3N X   1      44.110  20.070  39.990  0.00  0.00
ATOM    520  H28 C3N X   1      35.960  20.030  39.930  0.00  0.00
```

```
ATOM    521  H29 C3N X   1      24.690  26.550  40.070  0.00  0.00
ATOM    522  H30 C3N X   1      20.720  33.480  40.100  0.00  0.00
ATOM    523 N106 C3N X   1      32.690  21.760  39.920  0.00  0.00
ATOM    524 N107 C3N X   1      37.530  21.770  40.120  0.00  0.00
ATOM    525 N108 C3N X   1      42.440  21.780  40.020  0.00  0.00
ATOM    526 N109 C3N X   1      47.240  21.760  40.060  0.00  0.00
ATOM    527 N110 C3N X   1      20.570  42.810  39.970  0.00  0.00
ATOM    528 N111 C3N X   1      59.480  42.810  39.910  0.00  0.00
ATOM    529 N112 C3N X   1      22.990  46.990  39.920  0.00  0.00
ATOM    530 N113 C3N X   1      57.020  46.990  40.010  0.00  0.00
ATOM    531 N114 C3N X   1      25.460  51.240  39.860  0.00  0.00
ATOM    532 N115 C3N X   1      54.560  51.240  40.060  0.00  0.00
ATOM    533 N116 C3N X   1      27.860  55.440  39.950  0.00  0.00
ATOM    534 N117 C3N X   1      52.170  55.410  39.990  0.00  0.00
ATOM    535 N118 C3N X   1      27.810  24.570  40.060  0.00  0.00
ATOM    536 N119 C3N X   1      52.170  24.540  39.960  0.00  0.00
ATOM    537 N120 C3N X   1      25.410  28.760  40.000  0.00  0.00
ATOM    538 N121 C3N X   1      54.570  28.770  40.030  0.00  0.00
ATOM    539 N122 C3N X   1      22.990  32.990  40.020  0.00  0.00
ATOM    540 N123 C3N X   1      57.000  33.020  39.990  0.00  0.00
ATOM    541 N124 C3N X   1      20.610  37.160  39.930  0.00  0.00
ATOM    542 N125 C3N X   1      59.460  37.170  39.990  0.00  0.00
ATOM    543 N126 C3N X   1      32.740  58.270  40.000  0.00  0.00
ATOM    544 N127 C3N X   1      37.560  58.230  40.070  0.00  0.00
ATOM    545 N128 C3N X   1      42.440  58.240  39.950  0.00  0.00
ATOM    546 N129 C3N X   1      47.290  58.270  40.000  0.00  0.00
ATOM    547  H31 C3N X   1      32.830  59.260  40.040  0.00  0.00
ATOM    548  H32 C3N X   1      32.710  20.760  39.910  0.00  0.00
ATOM    549  H33 C3N X   1      37.540  20.790  40.040  0.00  0.00
ATOM    550  H34 C3N X   1      42.460  20.780  39.930  0.00  0.00
ATOM    551  H35 C3N X   1      47.160  20.760  39.990  0.00  0.00
ATOM    552  H36 C3N X   1      19.740  43.370  40.000  0.00  0.00
ATOM    553  H37 C3N X   1      60.330  43.340  39.900  0.00  0.00
ATOM    554  H38 C3N X   1      22.130  47.480  39.860  0.00  0.00
ATOM    555  H39 C3N X   1      57.880  47.480  39.990  0.00  0.00
ATOM    556  H40 C3N X   1      24.590  51.740  39.790  0.00  0.00
ATOM    557  H41 C3N X   1      55.430  51.730  40.110  0.00  0.00
ATOM    558  H42 C3N X   1      26.970  55.890  39.980  0.00  0.00
ATOM    559  H43 C3N X   1      53.070  55.850  40.000  0.00  0.00
ATOM    560  H44 C3N X   1      26.900  24.150  40.020  0.00  0.00
ATOM    561  H45 C3N X   1      53.060  24.070  39.940  0.00  0.00
ATOM    562  H46 C3N X   1      24.510  28.310  40.010  0.00  0.00
ATOM    563  H47 C3N X   1      55.460  28.300  40.010  0.00  0.00
ATOM    564  H48 C3N X   1      22.140  32.450  40.050  0.00  0.00
ATOM    565  H49 C3N X   1      57.850  32.490  40.070  0.00  0.00
ATOM    566  H50 C3N X   1      19.790  36.590  39.890  0.00  0.00
ATOM    567  H51 C3N X   1      60.280  36.590  39.990  0.00  0.00
ATOM    568  H52 C3N X   1      37.630  59.230  40.010  0.00  0.00
ATOM    569  H53 C3N X   1      42.400  59.250  39.950  0.00  0.00
ATOM    570  H54 C3N X   1      47.250  59.270  39.960  0.00  0.00
ATOM    571 C364 C3N X   1      50.910  22.440  39.920  0.00  0.00
ATOM    572 C365 C3N X   1      19.380  39.230  40.000  0.00  0.00
ATOM    573 C366 C3N X   1      49.710  58.230  39.970  0.00  0.00
ATOM    574 C367 C3N X   1      30.270  21.740  40.000  0.00  0.00
ATOM    575 C368 C3N X   1      60.710  40.710  39.980  0.00  0.00
ATOM    576 C369 C3N X   1      29.060  57.540  40.070  0.00  0.00
ATOM    577  H55 C3N X   1      51.770  21.940  39.880  0.00  0.00
ATOM    578  H56 C3N X   1      18.510  38.730  39.960  0.00  0.00
```

```
ATOM    579  H57 C3N X   1      49.710  59.210  39.930  0.00  0.00
ATOM    580  H58 C3N X   1      30.300  20.740  39.990  0.00  0.00
ATOM    581  H59 C3N X   1      61.560  41.230  39.970  0.00  0.00
ATOM    582  H60 C3N X   1      28.190  58.000  40.160  0.00  0.00
END
```
