# Supplementary material for: C3N nanodots inhibits Aβ peptides aggregation pathogenic path in Alzheimer’s disease
Source: Nat Commun. 2023 Sep 15;14:5718. doi: 10.1038/s41467-023-41489-y (PMC10504243; doi:10.1038/s41467-023-41489-y)
Supplement: Supplementary file 5 — Reporting Summary [file 41467_2023_41489_MOESM5_ESM.pdf]

nature portfolio

Corresponding author(s): Ruhong Zhou

Last updated by author(s): Aug 22, 2023

Reporting Summary

Nature Portfolio wishes to improve the reproducibility of the work that we publish. This form provides structure for consistency and transparency in reporting. For further information on Nature Portfolio policies, see our Editorial Policies and the Editorial Policy Checklist.

Statistics

For all statistical analyses, confirm that the following items are present in the figure legend, table legend, main text, or Methods section.

n/a	Confirmed	
☐	☒	The exact sample size (*n*) for each experimental group/condition, given as a discrete number and unit of measurement
☐	☒	A statement on whether measurements were taken from distinct samples or whether the same sample was measured repeatedly
☐	☒	The statistical test(s) used AND whether they are one- or two-sided<br>*Only common tests should be described solely by name; describe more complex techniques in the Methods section.*
☒	☐	A description of all covariates tested
☒	☐	A description of any assumptions or corrections, such as tests of normality and adjustment for multiple comparisons
☐	☒	A full description of the statistical parameters including central tendency (e.g. means) or other basic estimates (e.g. regression coefficient) AND variation (e.g. standard deviation) or associated estimates of uncertainty (e.g. confidence intervals)
☐	☒	For null hypothesis testing, the test statistic (e.g. *F*, *t*, *r*) with confidence intervals, effect sizes, degrees of freedom and *P* value noted<br>*Give P values as exact values whenever suitable.*
☒	☐	For Bayesian analysis, information on the choice of priors and Markov chain Monte Carlo settings
☒	☐	For hierarchical and complex designs, identification of the appropriate level for tests and full reporting of outcomes
☒	☐	Estimates of effect sizes (e.g. Cohen's *d*, Pearson's *r*), indicating how they were calculated

Our web collection on statistics for biologists contains articles on many of the points above.

Software and code

Policy information about availability of computer code

Data collection	Gen5, Living Image 4.4
Data analysis	Prism 9.0, Image J, origin 9.0

For manuscripts utilizing custom algorithms or software that are central to the research but not yet described in published literature, software must be made available to editors and reviewers. We strongly encourage code deposition in a community repository (e.g. GitHub). See the Nature Portfolio guidelines for submitting code & software for further information.

Data

Policy information about availability of data

All manuscripts must include a data availability statement. This statement should provide the following information, where applicable:

- Accession codes, unique identifiers, or web links for publicly available datasets
- A description of any restrictions on data availability
- For clinical datasets or third party data, please ensure that the statement adheres to our policy

The data that support the findings of this paper are available in the paper and supplementary information files.

Research involving human participants, their data, or biological material

Policy information about studies with human participants or human data. See also policy information about sex, gender (identity/presentation), and sexual orientation and race, ethnicity and racism.

Reporting on sex and gender	*Use the terms sex (biological attribute) and gender (shaped by social and cultural circumstances) carefully in order to avoid confusing both terms. Indicate if findings apply to only one sex or gender; describe whether sex and gender were considered in study design; whether sex and/or gender was determined based on self-reporting or assigned and methods used.*<br>*Provide in the source data disaggregated sex and gender data, where this information has been collected, and if consent has been obtained for sharing of individual-level data; provide overall numbers in this Reporting Summary. Please state if this information has not been collected.*<br>*Report sex- and gender-based analyses where performed, justify reasons for lack of sex- and gender-based analysis.*
Reporting on race, ethnicity, or other socially relevant groupings	*Please specify the socially constructed or socially relevant categorization variable(s) used in your manuscript and explain why they were used. Please note that such variables should not be used as proxies for other socially constructed/relevant variables (for example, race or ethnicity should not be used as a proxy for socioeconomic status).*<br>*Provide clear definitions of the relevant terms used, how they were provided (by the participants/respondents, the researchers, or third parties), and the method(s) used to classify people into the different categories (e.g. self-report, census or administrative data, social media data, etc.)*<br>*Please provide details about how you controlled for confounding variables in your analyses.*
Population characteristics	*Describe the covariate-relevant population characteristics of the human research participants (e.g. age, genotypic information, past and current diagnosis and treatment categories). If you filled out the behavioural & social sciences study design questions and have nothing to add here, write "See above."*
Recruitment	*Describe how participants were recruited. Outline any potential self-selection bias or other biases that may be present and how these are likely to impact results.*
Ethics oversight	*Identify the organization(s) that approved the study protocol.*

Note that full information on the approval of the study protocol must also be provided in the manuscript.

Field-specific reporting

Please select the one below that is the best fit for your research. If you are not sure, read the appropriate sections before making your selection.

- [x] Life sciences
- [ ] Behavioural & social sciences
- [ ] Ecological, evolutionary & environmental sciences

For a reference copy of the document with all sections, see nature.com/documents/nr-reporting-summary-flat.pdf

Life sciences study design

All studies must disclose on these points even when the disclosure is negative.

Sample size	No statistical methods were used to pre-determine sample sizes but our sample sizes are similar to those reported in previous publications.
Data exclusions	No samples were excluded from the study.
Replication	All experiments were performed with at least three independent biological samples. All results relevant to the replicated trials were obtained independently for reliability. All attempts at replication were successful.
Randomization	The animals were littermates, and inbred lines were used, where the individual mice were identical, therefore no specific randomization was needed. Mice were grouped according to genotype before they were randomly assigned to the experimental conduct.
Blinding	All researchers performing animal experiments and/or data analysis were blinded. However, cell culture treatments and analyses were mostly performed by the same individual, so blinding was not always possible. Wherever possible, a second researcher blinded to the analysis confirmed the result.

Reporting for specific materials, systems and methods

We require information from authors about some types of materials, experimental systems and methods used in many studies. Here, indicate whether each material, system or method listed is relevant to your study. If you are not sure if a list item applies to your research, read the appropriate section before selecting a response.

Materials & experimental systems

n/a	Involved in the study
	☒ Antibodies
	☒ Eukaryotic cell lines
☒	Palaeontology and archaeology
	☒ Animals and other organisms
☒	Clinical data
☒	Dual use research of concern
☒	Plants

Methods

n/a	Involved in the study
☒	ChIP-seq
☒	Flow cytometry
☒	MRI-based neuroimaging

Antibodies

Antibodies used

All antibodies used in the study are described in the Methods section in detail, which include the catalogue number, dilution factor, providers and etc.
Amyliod Fibril-Conformation-Specific (mOC87, abcam, Cat#: ab201062, 1:8000);
SNAP25 (Synaptic systems, Cat#: 111-002, 1:2,000);
VAMP2 (abcam, Cat#: ab3347, 1:1,000);
β-actin (4D3, Bioworld Technology, Cat#: BS6007M, 1:5000);
Purified anti-β-Amyloid 1-16 (6E10, Covance, SIG-39320, 1:500);
MAP2 (AP20, Millipore, Cat#: MAB3418, 1:1,000);
Peroxidase affinipure donkey anti-rabbit IgG (H + L) (#711-035-152, Jackson ImmunoResearch, 1:10000);
Peroxidase affinipure donkey anti-mouse IgG (H + L) (#715-035-151, Jackson ImmunoResearch, 1:10000);
Cy3 affinipure donkey anti-mouse IgG (H + L) (#715-165-151, 1:400);
Alexa Fluor® 488 affinipure donkey anti-rabbit IgG (H + L) (#711-545-152, 1:400).

Validation

Antibodies specific for the required antigens/epitopes were purchased from commercial sources

Eukaryotic cell lines

Policy information about cell lines and Sex and Gender in Research

Cell line source(s)

rat adrenal pheochromocytoma cells (PC12, CRL-1721), human umbilical vein endothelial cells (HUVCEs, PCS-100-013) and human neuroblastoma cells (sh-sy5y, CRL-2266) lines were purchased by American Type Culture Collection (ATCC).
mouse brain microvascular endothelial cells (bEnd.3, cl-0598) and BV2 murine microglial cells (BV2, cl-0493,bEnd.3 cell lines were purchased from Procell.
Mouse primary cortical neurons were obtained from embryonic day 18 C57BL/6J mice.
Primary astrocyte cultures were extracted from the cerebral cortex of 1-3-d-old rats.

Authentication

None of the lines used were authenticated.

Mycoplasma contamination

Cell lines were not tested for Mycoplasma contamination.

Commonly misidentified lines
(See ICLAC register)

No commonly misidentified cell lines were used in the study.

Animals and other research organisms

Policy information about studies involving animals; ARRIVE guidelines recommended for reporting animal research, and Sex and Gender in Research

Laboratory animals

For the C3N treatment APP/PSl [B6C3-Tg (APPswePSEN1dE9)/Nju] double transgenic AD mice and C57BL/6 WT mice were purchased from Nanjing Model Animal Research Center. C57BL/6J female mice were crossed with APP/PS1 male mice. Mice were housed in a SPF-grade animal facility withwith free access to chow and drinking water under a 12-hour light/dark cycle under constant temperature (22 ± 1°C) and humidity (40–70%).

Wild animals

Our experiments did not used wild animals .

Reporting on sex

Male mice were used in our experiment.

Field-collected samples

This study did not involve field collected samples.

Ethics oversight

All animal protocols were approved by the Animal Ethics Committee of Soochow University (protocol no. SUDA201807A422, SUDA201907A025 and SUDA202007A648) and were in compliance with relevant ethical regulations.

Note that full information on the approval of the study protocol must also be provided in the manuscript.
